# Development of a Multidomain Conceptual Framework for Frozen Shoulder: A Systematic Review Integrating Clinical, Biological, Psychological and Lifestyle-Related Contributors

**DOI:** 10.3390/jcm15176684

**Published:** 2026-08-28

**Authors:** Santiago Navarro-Ledesma, José Javier Pérez-Montilla, Dina Hamed-Hamed, Fabrizio Brindisino, Filip Struyf

**Affiliations:** 1 Department of Physical Therapy, Faculty of Health Sciences, Campus of Melilla, University of Granada, Querol Street 5, 52004 Melilla, Spain; perezmontilla@ugr.es; 2 Clinical Medicine and Public Health PhD Program, Faculty of Health Sciences, University of Granada, 18061 Granada, Spain; dhamed@correo.ugr.es; 3 Hospital Universitario de Melilla, C/Luis de Ostáriz 12, 52005 Melilla, Spain; 4 Department of Medicine and Health Science “Vincenzo Tiberio”, University of Molise, 86100 Campobasso, Italy; fabrizio.brindisino@unimol.it; 5 Research Group MOVANT, Department of Rehabilitation Sciences and Physiotherapy, Faculty of Medicine and Health Sciences, University of Antwerp, 2610 Antwerp, Belgium; filip.struyf@uantwerpen.be

**Keywords:** adhesive capsulitis, phenotype, biomarkers, disease progression, prognosis

## Abstract

**Background/Objectives**: Frozen shoulder (FS) is increasingly recognised as a heterogeneous condition in which clinical presentation, metabolic/endocrine burden, imaging phenotype, psychological factors, lifestyle-related contributors, and inflammatory biology may all contribute to disease expression and outcome. However, these contributors have not previously been integrated within a unified multidomain framework for whole-person phenotyping. To develop an evidence-derived multidomain conceptual framework for FS by integrating clinical, biological, psychological, and lifestyle-related contributors into a clinically interpretable structure. **Methods**: A systematic review with quantitative evidence synthesis was conducted following methodological principles for systematic reviews and reported in accordance with PRISMA 2020. The protocol was registered in PROSPERO (CRD420251158406). Observational studies relevant to predefined FS domains were identified, classified according to analytic contribution, and synthesised using a structured evidence-weighted approach. Where quantitative pooling was methodologically appropriate, meta-analytic synthesis was undertaken; where heterogeneity precluded conventional pooling, evidence was integrated through structured domain-level quantitative synthesis. Within-sphere and cross-sphere weights were derived from directness, consistency, prognostic or phenotyping relevance, methodological robustness, risk-of-bias profile, and domain specificity, and should be interpreted as exploratory evidence-derived estimates. **Results**: The final multidomain corpus comprised 63 studies, including 39 allocated to the clinical sphere, 5 to the metabolic/endocrine sphere, 10 to the psychological sphere, 8 to the imaging sphere, and 1 to the inflammatory sphere as direct but lower-confidence support. The final exploratory framework retained five spheres: clinical, metabolic/endocrine, imaging, psychological, and inflammatory. Within-sphere weighting identified baseline stiffness/range-of-motion restriction and symptom duration as dominant clinical components, diabetes-related burden and glycaemic control as dominant metabolic/endocrine components, capsular structural biomarkers as dominant imaging components, distress/mood together with sleep-related burden as dominant psychological components, and IL-1β-related biomarker and susceptibility evidence as the principal retained inflammatory signal. Provisional cross-sphere weighting yielded global contributions of 24.0% for the metabolic/endocrine sphere, 22.9% for the imaging sphere, 21.1% for the clinical sphere, 20.0% for the psychological sphere, and 12.0% for the inflammatory sphere. **Conclusions**: FS appears to be more appropriately represented as a weighted multidomain condition than purely as a clinical stiffness syndrome. The identified domains should not be interpreted as isolated entities, but rather as interacting and overlapping contributors within a whole-person clinical presentation. The proposed framework should be interpreted as an initial conceptual platform for future validation and personalised treatment-allocation research rather than as a clinically validated decision-support tool.

## 1. Introduction

Frozen shoulder (FS) is traditionally characterised by spontaneous or progressive shoulder pain, marked limitation of glenohumeral motion, and substantial disability [1,2]. Although it is often approached clinically as a recognisable stiffness syndrome, its real-world presentation is clinically and biologically heterogeneous [3]. Patients differ not only in pain intensity, irritability, and degree of mobility loss, but also in endocrine–metabolic comorbidity, psychological burden, sleep disturbance, imaging phenotype, lifestyle-related factors, and response to treatment [4]. Therefore, a purely symptom-based, range-of-motion-based, or localised capsular interpretation is unlikely to capture the full complexity of FS, in which biological, metabolic, behavioural, psychological, inflammatory, and lifestyle-related factors may interact in shaping clinical presentation and recovery trajectory [3,4,5].

Epidemiologically, FS represents a relevant clinical burden. Most contemporary summaries estimate its frequency in the general population at approximately 2–5%, with predominance in women and individuals between 40 and 70 years of age [6,7], although lower estimates have been reported in some primary-care datasets depending on case definition and sampled population [8]. The epidemiological signal is stronger in systemic-risk groups, as FS is substantially more frequent in individuals with diabetes mellitus (DM) and thyroid disease, with reported prevalence estimates of approximately 10–38% in these populations [9,10]. This supports the view that FS should not be understood solely as an isolated local shoulder disorder.

This multidomain heterogeneity has important practical consequences. Treatment selection is still frequently guided by broad tissue irritability categories rather than by a structured estimate of which clinical or non-clinical domains are most relevant in each patient [5,11]. Consequently, two patients with apparently similar range-of-motion restrictions may differ substantially in the degree to which their presentation is influenced by metabolic burden, tissue-level features, psychological distress, sleep-related symptom amplification, lifestyle-related factors, movement avoidance, or active inflammatory biology [12,13,14,15]. This creates a gap between the multidimensional nature of FS and the relatively non-quantified way in which treatment is commonly prioritised [15].

Several lines of evidence support a multidomain interpretation of FS. Clinical studies have shown that baseline stiffness, symptom duration, pain intensity, and disease stage are associated with clinical trajectory and recovery [16]. Endocrine–metabolic comorbidities, particularly DM and poorer glycaemic control, have been associated with recurrence, poorer treatment response, and less favourable long-term outcome [9,14,15]. MRI and ultrasound studies have identified structural capsular abnormalities, edema-related activity signs, and vascular imaging features with potential relevance for phenotype, stage, or prognosis [17,18]. Psychological distress, pain-related cognitive factors, psychosocial quality of life, and sleep-related burden appear to modulate symptom severity and perceived recovery [19,20,21]. Finally, inflammatory and fibro-inflammatory mechanisms have been implicated in FS pathobiology through cytokine activity, matrix remodelling, and capsular fibrosis [22].

Previous multimodal and biopsychosocial approaches have helped move the field beyond a purely local capsular model, but important methodological gaps remain. Existing models recognise interactions between clinical presentation, systemic biological factors, imaging findings, psychological burden, and inflammatory mechanisms; however, these contributors have rarely been organised into a structured framework that can be applied at patient level. Sleep-related burden remains underrepresented, imaging evidence is often interpreted through single-modality perspectives, and the relative contribution of each domain has not been clearly operationalised within a unified model [4,14,15,23]. Therefore, published work has not yet fully progressed from multidomain description to a framework capable of supporting sphere-level interpretation and future personalised treatment-allocation research [4,16].

The present study was designed to address this gap through two linked but distinct phases. First, we conducted a systematic review with quantitative evidence synthesis to identify and organise evidence related to clinical, metabolic/endocrine, imaging, psychological, inflammatory, and lifestyle-related contributors to FS. Second, we used this evidence base to develop an exploratory multidomain conceptual framework and research-use operational prototype for patient-level phenotyping. The aim was not to provide a validated clinical decision tool, but to create an evidence-derived structure capable of organising heterogeneous research into clinically interpretable domains and estimating their provisional relative contributions. Accordingly, the framework is intended to support whole-person reasoning and to provide a first research platform for future validation, recalibration, and personalised treatment-allocation studies.

## 2. Methods

### 2.1. Study Design and Reporting Framework

This study was designed as a systematic review with quantitative evidence synthesis followed by exploratory multidomain conceptual framework development. The review was conducted following methodological principles for systematic reviews, reported in accordance with PRISMA 2020 [24], and prospectively registered in PROSPERO (registration ID: CRD420251158406). Methodological principles relevant to observational prognostic research were used to guide evidence interpretation, while STROBE/TRIPOD-aligned logic informed the framework-development and prototype-operationalisation components.

The work comprised two linked but distinct phases. Phase 1 consisted of systematic evidence identification, study selection, data extraction, risk-of-bias appraisal, and domain-level evidence synthesis. Phase 2 consisted of conceptual framework development, in which the evidence identified in Phase 1 was organised into clinically interpretable domains and translated into a research-use operational prototype. This separation was introduced to distinguish conclusions directly supported by the reviewed literature from the interpretative framework-development process.

Quantitative pooling was undertaken only when the retained studies were sufficiently comparable in design, population, predictor, outcome, and effect metric to permit meta-analytic synthesis. When conventional pooling was not methodologically appropriate, evidence was integrated using structured domain-level quantitative synthesis. Because the primary objective was multidomain framework development rather than estimation of a single pooled effect or validation of a prediction model, all domain weights and prototype scores were treated as exploratory and provisional. No internal validation, external validation, calibration analysis, reproducibility testing, or predictive-performance analysis of the prototype was performed in the present study.

### 2.2. Search Strategy and Source Selection

The literature base was assembled through structured domain-focused searches designed to identify studies relevant to predefined FS domains: clinical presentation and prognosis, metabolic/endocrine burden, imaging biomarkers, psychological constructs, sleep-related burden, and inflammatory biology. Because the project involved framework development, domain-specific searching was refined when conceptual gaps became apparent during synthesis, particularly in relation to sleep-related burden and the expansion of the imaging domain from an initial ultrasound-centred formulation to a combined MRI-plus-ultrasound domain.

Comprehensive literature searches were performed in PubMed, Web of Science, and ScienceDirect on 12 January 2026. Searches were limited to studies published within the previous ten years, written in English, involving human adults, and available as open access full text. The search strategy relied primarily on free-text terms combined with Boolean operators and applied to title/abstract or topic fields depending on database functionality. The complete search strategies, fields, and filters used for each database are reported in Table 1.

### 2.3. Study Selection and Eligibility Criteria

Two reviewers (S.N.L. and J.J.P.M.) independently screened titles and abstracts, followed by full-text assessment of potentially eligible reports. Disagreements were resolved through discussion and consensus. Studies were eligible when they investigated frozen shoulder or adhesive capsulitis in adults and reported extractable quantitative data relevant to at least one predefined domain of interest. Eligible designs included prospective and retrospective cohorts, observational prognostic studies, case–control studies, cross-sectional studies, imaging studies, biomarker studies, and non-randomised interventional studies when they provided extractable phenotype-, prognosis-, treatment-response-, or domain-relevant data.

Randomised trials, reviews, editorials, conference abstracts without sufficient quantitative detail, single-patient case reports, and purely narrative publications were not included in the core weighting framework. Selected non-core studies could be retained only when they provided contextual biological or conceptual support and were explicitly labelled as non-core evidence. Studies were excluded when they did not investigate FS specifically, lacked extractable quantitative data relevant to the predefined domains, or focused on structurally non-comparable clinical scenarios without sufficient interpretability for the framework.

### 2.4. Analytic Classification

After full-text review, studies were classified according to their analytic contribution. The full corpus comprised all studies mapped to one of the final domains. A smaller core analytic subset was then identified to inform direct weighting when the study design, population, predictor, and outcome were considered sufficiently relevant and interpretable for domain-level synthesis. Studies that were clinically informative but less directly comparable were retained for supportive, sensitivity, or contextual interpretation rather than weighted equivalently with the core analytic subset. This two-step classification was used to reduce structural heterogeneity and avoid pooling or weighting evidence with substantially different analytic meaning.

### 2.5. Domain Definition and Predictor Reassignment

The final framework retained five domains: clinical, metabolic/endocrine, imaging, psychological, and inflammatory. Sleep was not retained as an independent sixth domain; after targeted review of the FS sleep literature, sleep-related burden was incorporated as a formal subdomain within the psychological domain because of its conceptual overlap with distress, pain-related burden, irritability, and psychosocial functioning. MRI and ultrasound were integrated into a single imaging domain because the retained evidence converged on related structural, vascular, and activity-related tissue features rather than on independent modality-specific constructs.

To reduce double counting, predictors were assigned according to their principal biological or clinical meaning rather than according to the nominal focus of the source paper. For example, diabetes mellitus, HbA1c, thyroid disease, dyslipidaemia, and liver-associated metabolic markers were assigned to the metabolic/endocrine domain even when reported in clinically oriented studies. Conversely, baseline pain, symptom duration, stage/irritability, disability, range-of-motion restriction, and autonomic symptoms were retained within the clinical domain. Domain assignment and predictor reassignment rules are provided in Supplementary File S2.

### 2.6. Data Extraction

Data extraction was performed at the study and domain levels. Study-level extraction captured design, population characteristics, sample size, FS subtype or treatment context, follow-up duration, outcomes, candidate predictors, and analytic role within the framework. Domain-level extraction captured predictor identity, linked outcome, direction of association, effect metric, adjustment status, prognostic versus cross-sectional relevance, methodological interpretability, and relevance to the target domain. During prototype operationalisation, an additional extraction layer identified candidate patient-level inputs, preferred scales, severity direction, standardisation rules, and missing-data handling strategies. Extraction was performed by J.J.P.M. and independently checked by S.N.L.; discrepancies were resolved by consensus.

### 2.7. Risk of Bias and Methodological Quality

Risk of bias and methodological quality were assessed using design-specific tools. ROBINS-I was used for non-randomised interventional studies where applicable; QUIPS for prognostic studies; NIH Study Quality Assessment Tools and Joanna Briggs Institute tools for observational, cross-sectional, imaging, biomarker, and genetic-association studies; and JBI criteria for case-level contextual evidence when applicable. Because the retained literature was heterogeneous across domains, risk of bias was not applied as a universal exclusion threshold. Instead, it informed confidence in domain interpretation, subdomain weighting, and cross-domain weighting. Detailed risk-of-bias and methodological quality assessments are provided in Supplementary File S1.

### 2.8. Evidence Synthesis and Weighting Procedure

Each domain was synthesised using a structured evidence-weighted approach. Priority was given to longitudinal adjusted prognostic evidence, followed by longitudinal unadjusted evidence, treatment-response or phenotype-relevant observational evidence, and cross-sectional or subgroup evidence. Within each domain, predictors were grouped into clinically meaningful subdomains. The provisional weighting process considered the same criteria across domains: directness of evidence, consistency across retained studies, prognostic or phenotyping relevance, methodological robustness, risk-of-bias profile, specificity of the construct to the domain, and operational interpretability.

The weighting procedure was not a data-driven coefficient estimation from a single empirical model. Rather, it was a structured reviewer-judgement process applied consistently across domains. For each domain, subdomains were ranked and assigned provisional internal weights according to the strength and interpretability of the retained evidence, and weights were normalised so that the internal subdomains summed to 100% within each domain. Cross-domain weights were then assigned using the same evidence-appraisal principles and normalised to 100% across the five domains. The internal weighting structure is provided in Supplementary File S2, and the main quantitative signals supporting weighting decisions are summarised in Supplementary File S3.

### 2.9. Prototype Operational Scoring Model

To translate the conceptual framework into a research-use operational prototype, each domain was expressed as a weighted combination of standardised subdomain inputs. The provisional multidomain equation was defined as: FS multidomain burden score = 0.211 (C) + 0.240 (M) + 0.229 (Im) + 0.200 (P) + 0.120 (Inf), where C represents the clinical domain, M the metabolic/endocrine domain, Im the imaging domain, P the psychological domain, and Inf the inflammatory domain. These coefficients correspond to the provisional cross-domain weights derived from the structured evidence-weighting procedure.

One principal operational input was selected for each construct whenever possible to preserve interpretability. In the clinical domain, movement restriction was represented by external rotation, forward flexion, and abduction deficits; disability by SPADI total; and pain by a 0–10 VAS/NRS measure. In the metabolic/endocrine domain, diabetes mellitus and HbA1c were selected as principal drivers. In the imaging domain, structural capsular biomarkers and activity-related imaging signs were prioritised. In the psychological domain, distress/mood and sleep-related burden were operationalised using HADS- and PSQI-based constructs. In the inflammatory domain, IL-1β-related biomarker activity and inflammatory susceptibility were retained as provisional inputs. The complete operational matrix is provided in Supplementary File S4.

All operational inputs were standardised to a 0–100 scale before weighting. Binary variables were coded as 0 or 100, continuous variables were linearly rescaled to predefined ranges, and ordinal variables were mapped to equally spaced 0–100 categories. For range-of-motion variables, severity was defined using movement deficit rather than raw mobility values so that higher scores consistently represented greater burden. This standardisation was used to permit prototype calculation and should not be interpreted as evidence that all relationships between predictors and disease burden are linear.

### 2.10. Handling of Missing Data and Sensitivity Specification

When one input was missing within a subdomain, available inputs in that subdomain were proportionally reweighted. If an entire subdomain was unavailable, the remaining subdomains within the corresponding domain were rescaled to sum to 1, with reduced-confidence interpretation. Because the inflammatory domain was supported by substantially less direct retained evidence and is less routinely measurable in clinical settings, a four-domain sensitivity specification was also calculated by omitting the inflammatory domain and renormalising the remaining clinical, metabolic/endocrine, imaging, and psychological weights to 100%.

### 2.11. Worked Example

A realistic fictional patient profile was used to demonstrate implementation of the prototype. This worked example was included only to illustrate the calculation, operational feasibility, and presentation of a domain-specific profile. It was not used as validation evidence, and the resulting numerical values were not interpreted as having established diagnostic, prognostic, or treatment-allocation meaning.

## 3. Results

### 3.1. Search and Study Selection

**Figure 1:**
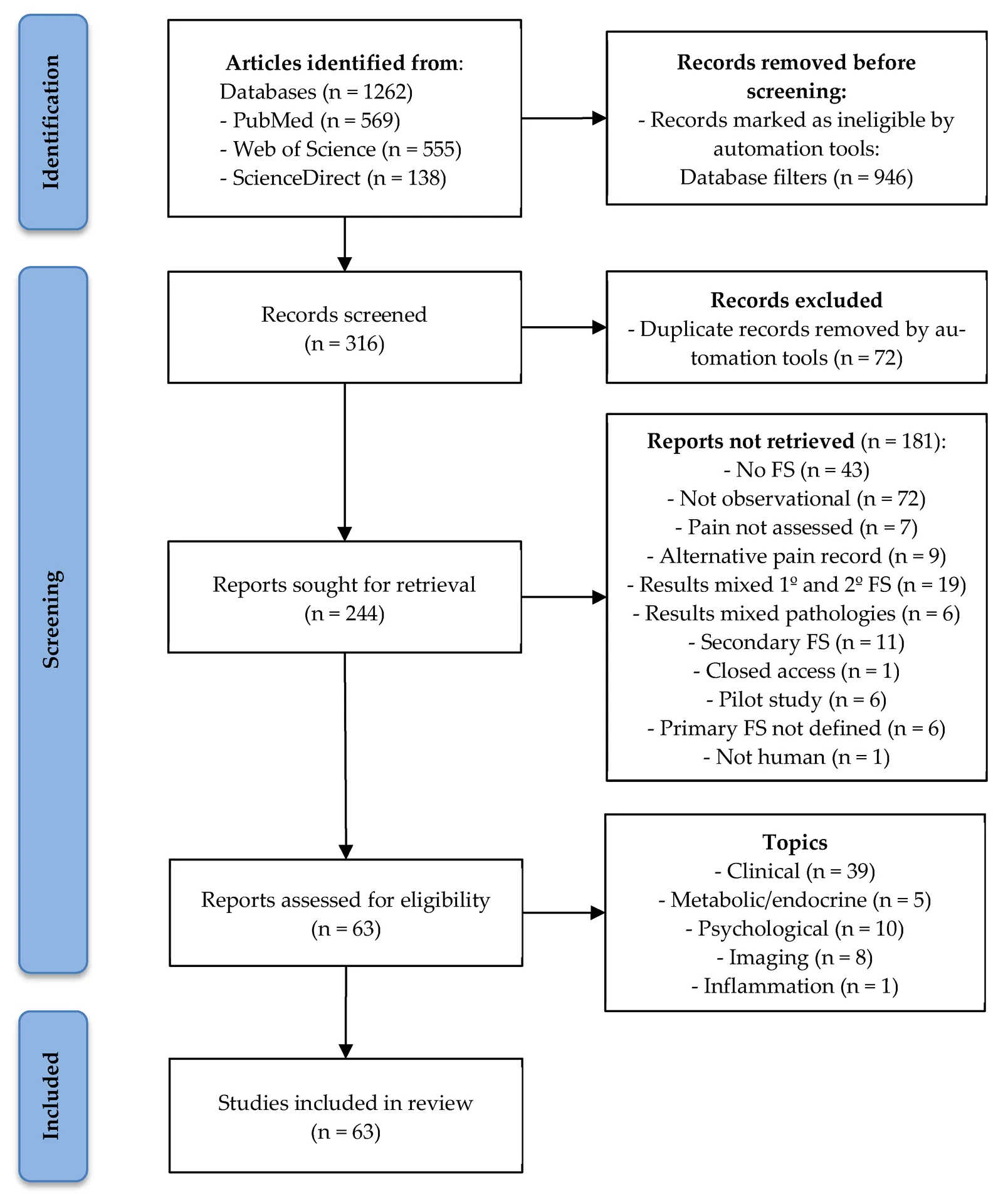
PRISMA flow diagram.

The study selection process and reasons for exclusion are shown in the PRISMA flow diagram (Figure 1). The database search identified 1262 records across PubMed, Web of Science, and ScienceDirect. After filters were applied, 316 records remained: PubMed (n = 74), Web of Science (n = 209), and ScienceDirect (n = 33). Following the removal of 72 duplicates, 244 records were screened by title and abstract. Of these, 181 were excluded, leaving 63 studies for inclusion in the review.

The final multidomain corpus comprised 63 studies including 8648 participants. Thirty-nine studies were allocated to the clinical domain, five to the metabolic/endocrine domain, ten to the psychological domain, eight to the imaging domain, and one to the inflammatory domain. The core analytic subset directly informing weighting was smaller and comprised 18 clinical studies, four metabolic/endocrine studies, six psychological studies, and seven imaging studies. The inflammatory domain was retained as a distinct but lower-confidence domain, supported by one direct case-control biomarker/genetic-susceptibility study and contextual biological evidence rather than by a comparably broad observational core. This imbalance in evidence volume and certainty was considered when interpreting the weighting structure.

### 3.2. Study Characteristics

Study characteristics by domain are summarised in Table 2. The clinical domain was the largest and most heterogeneous, including prospective and retrospective cohorts, comparative observational studies, non-randomised intervention cohorts, and prognostic studies evaluating pain, range of motion, disability, symptom duration, treatment response, recurrence, or recovery trajectory. The metabolic/endocrine domain included studies addressing diabetes mellitus, glycaemic control, thyroid/endocrine variables, dyslipidaemia, and liver-associated metabolic markers. The imaging domain included MRI- and ultrasound-based studies evaluating capsular thickness, coracohumeral ligament abnormalities, axillary recess findings, edoema or hyperintensity, enhancement, and vascular flow. The psychological domain included studies addressing anxiety, depression, psychosocial quality of life, self-efficacy, kinesiophobia, and sleep-related burden. The inflammatory domain included limited direct biomarker and susceptibility evidence.

### 3.3. Risk of Bias and Methodological Quality

Risk-of-bias assessment showed that the retained evidence was methodologically heterogeneous and largely observational, with most studies showing at least moderate concerns. In non-randomised interventional studies assessed with ROBINS-I, common sources of bias included confounding, absence of randomisation, selection of specific patient populations, retrospective design, and incomplete use of appropriate control groups. Prognostic studies assessed with QUIPS frequently showed moderate-to-high concerns related to insufficient adjustment for confounders, heterogeneity in outcome definitions, and variability in statistical analysis and reporting. Studies evaluated with NIH and JBI tools generally showed moderate methodological quality, with recurrent limitations including limited confounding control, lack of longitudinal follow-up, and restricted ability to support causal inference. Detailed assessments are provided in Supplementary File S1.

At domain level, the clinical evidence base was the largest but also the most heterogeneous. The metabolic/endocrine and imaging domains were supported by smaller but more conceptually coherent evidence sets, although limitations remained in external generalisability, confounding control, imaging protocol standardisation, and biomarker assessment. The psychological domain relied substantially on self-reported constructs, which may increase measurement overlap between pain, disability, sleep disturbance, and psychological burden. The inflammatory domain carried the greatest interpretive uncertainty because the retained direct evidence was limited to a single case–control study, despite biological plausibility and relevance to FS pathophysiology.

### 3.4. Final Multidomain Architecture

**Figure 2:**
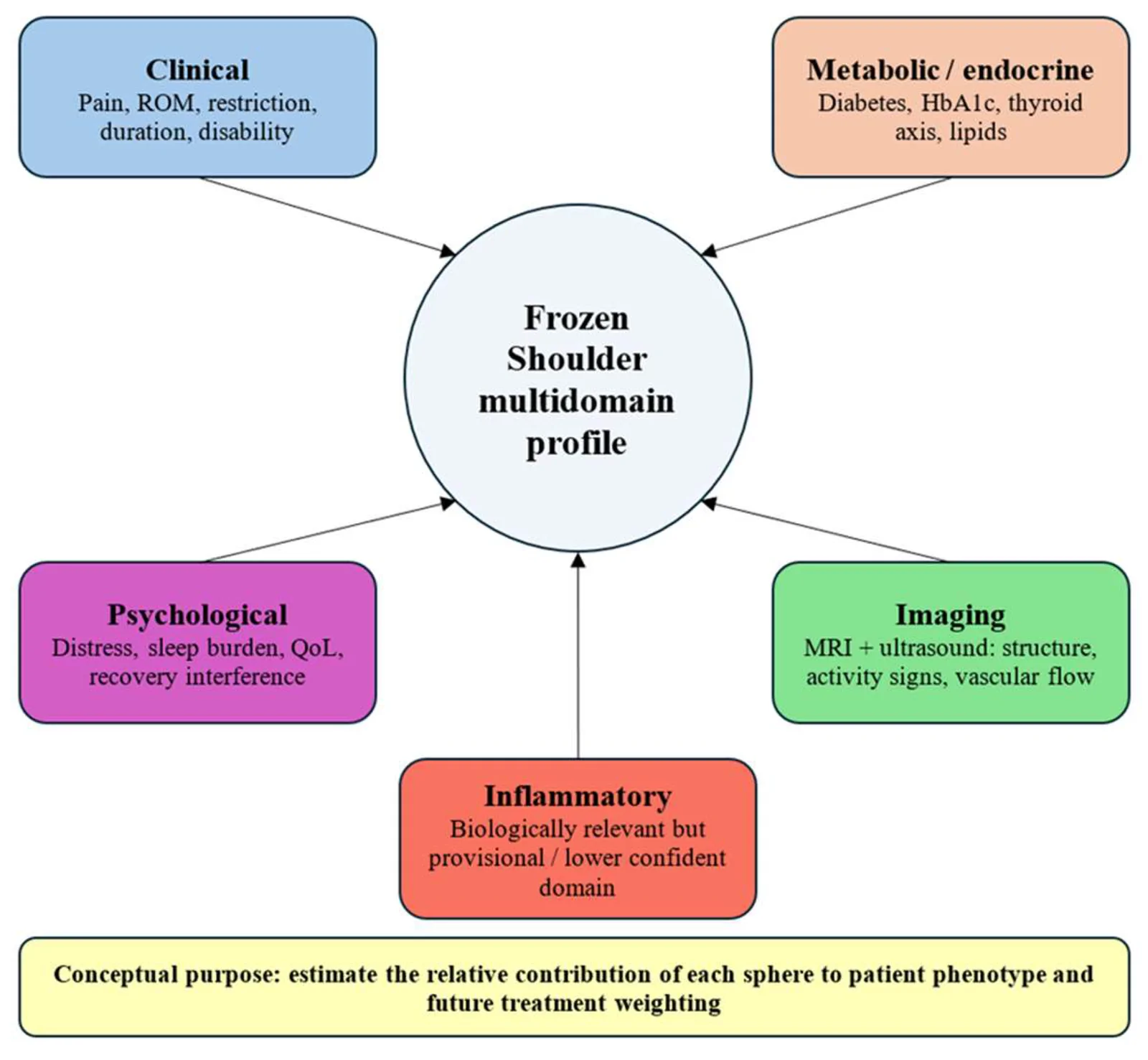
Conceptual architecture of the five-sphere multidomain framework for frozen shoulder. The figure illustrates the conceptual organisation of the framework rather than the final weighting structure. Note: Sleep-related burden is nested within the psychological sphere; MRI and ultrasound are integrated with a single imaging sphere. Acronyms: ROM, range of motion; QoL, Quality of Life; MRI, Magnetic Resonance Imaging; HbA1c, Glycated Haemoglobin.

The final multidomain architecture retained five domains: clinical, metabolic/endocrine, imaging, psychological, and inflammatory (Figure 2). Sleep-related burden was nested within the psychological domain because the retained evidence indicated overlap with distress, irritability, pain-related burden, and psychosocial functioning. MRI and ultrasound evidence were integrated into a single imaging domain because the studies converged on related structural and activity-related tissue features rather than on independent modality-specific constructs. Domain assignment and predictor reassignment rules are summarised in Supplementary File S2.

### 3.5. Domain-Level Evidence Synthesis

Within the clinical domain, the strongest recurrent signals were baseline stiffness/range-of-motion restriction and symptom duration. Baseline pain, stage/irritability, disability/function, autonomic symptoms, and contextual variables provided additional but smaller signals. The clinical evidence suggested that pain and mobility may follow different temporal trajectories, with pain often improving earlier and range-of-motion gains continuing over later follow-up windows.

Within the metabolic/endocrine domain, diabetes-related burden and glycaemic control were the dominant signals. Diabetes mellitus repeatedly emerged as an adverse factor associated with poorer treatment response, recurrence or reintervention, and less favourable long-term outcome. HbA1c and glucose-related measures suggested that glycaemic severity may provide information beyond binary diabetes status. Thyroid/endocrine-axis variables, lipid metabolism, and liver-associated metabolic markers contributed secondary exploratory signals.

Within the imaging domain, capsular structural biomarkers were the strongest internal component, including coracohumeral ligament thickening, inferior glenohumeral ligament-related findings, axillary recess capsule thickness, and ultrasound-defined capsular thickening patterns. Edoema-, hyperintensity-, and enhancement-related activity signs contributed a second layer, while microvascular imaging and compensatory sonographic features provided additional supportive signals.

Within the psychological domain, distress/mood was the strongest internal component, followed by sleep-related burden. Psychosocial quality of life and recovery-interference constructs, including self-efficacy and kinesiophobia, provided additional support. This structure indicates that the psychological domain extends beyond anxiety and depression alone and includes sleep-related and recovery-related constructs relevant to FS burden.

The inflammatory domain was retained because the included case–control evidence reported higher serum IL-1beta levels in patients with primary FS than in controls and an association between IL-1beta polymorphism and susceptibility. However, the breadth of direct retained evidence was substantially weaker than for the other domains. Therefore, the inflammatory domain was treated as biologically relevant but quantitatively provisional and lower confidence.

The provisional within-domain weighting structure is provided in Supplementary File S2. The main quantitative signals supporting domain interpretation and weighting are summarised in Supplementary File S3.

### 3.6. Provisional Cross-Domain Weighting

Cross-domain normalisation yielded provisional global weights of 24.0% for the metabolic/endocrine domain, 22.9% for the imaging domain, 21.1% for the clinical domain, 20.0% for the psychological domain, and 12.0% for the inflammatory domain. These values should be interpreted as evidence-derived exploratory weights rather than validated predictive coefficients. The relatively high clinical evidence volume did not translate into a dominant clinical weight because methodological heterogeneity, predictor overlap, and reassignment of endocrine–metabolic variables reduced its relative specificity. Conversely, the inflammatory domain was weighted more conservatively because its direct retained clinical evidence was limited.

### 3.7. Sensitivity Specification Excluding the Inflammatory Domain

Because the inflammatory domain was lower confidence and less operationally mature, a four-domain sensitivity specification was calculated by omitting the inflammatory domain and renormalising the remaining weights. In this sensitivity specification, the renormalised weights were 27.3% for the metabolic/endocrine domain, 26.0% for the imaging domain, 24.0% for the clinical domain, and 22.7% for the psychological domain. Applied to the worked example, the total multidomain score changed from 59.8/100 in the five-domain model to 60.7/100 in the four-domain sensitivity specification. This analysis was included to illustrate numerical stability of the worked example under omission of the least certain domain, not to validate the model.

### 3.8. Prototype Operationalisation

A prototype operational scoring matrix was developed to convert the conceptual framework into measurable patient-level inputs. For each domain and subdomain, the matrix defined the preferred operational variable, preferred scale or format, direction of severity, standardisation rule to a 0–100 metric, and missing-data handling strategy. The principal operational inputs are summarised in Supplementary File S4.

Within the clinical domain, movement restriction was operationalised using external rotation, forward flexion, and abduction deficits. Symptom duration, baseline pain, SPADI total, stage/irritability, autonomic symptoms, and contextual variables were retained as complementary clinical inputs. Within the metabolic/endocrine domain, diabetes mellitus and HbA1c were selected as principal operational drivers, with additional modifiers for thyroid dysfunction, lipid burden, and liver-associated metabolic abnormalities. Within the imaging domain, structural capsular biomarkers and activity-related imaging signs were prioritised. Within the psychological domain, HADS and PSQI were selected as the principal operational measures for distress and sleep, respectively. Within the inflammatory domain, IL-1beta-related biomarker activity and inflammatory susceptibility were retained as provisional inputs, although this pathway was considered lower confidence and less operationally mature because the retained direct evidence was limited and biomarker availability is not routine in most clinical FS settings.

### 3.9. Worked Example of Prototype Application

A realistic fictional patient profile was constructed to illustrate prototype implementation using movement deficits, symptom duration, pain intensity, disability, diabetes status, glycaemic control, imaging biomarkers, psychological scores, sleep-related burden, and provisional inflammatory inputs. This example was intended only to demonstrate calculation and presentation of a domain-specific profile. It was not used as validation evidence, and its numerical output should not be interpreted as having established diagnostic, prognostic, or treatment-allocation meaning.

**Figure 3:**
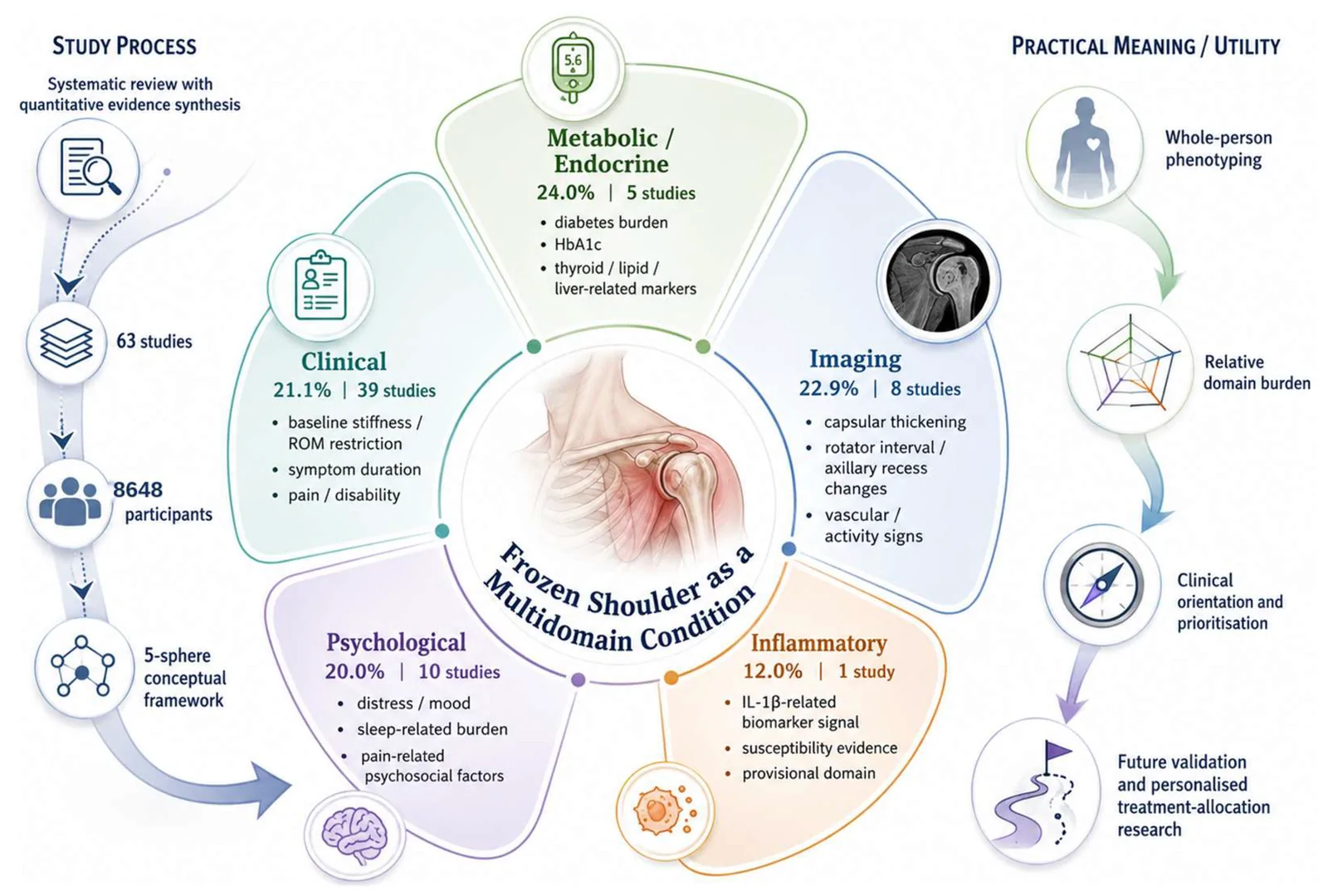
Visual synthesis of the evidence-derived multidomain framework for frozen shoulder. The figure summarises the five retained domains—clinical, metabolic/endocrine, imaging, psychological, and inflammatory, and their provisional relative contribution within the research-use framework. The model illustrates frozen shoulder as an interacting multidomain condition rather than as a purely local capsular stiffness syndrome. Percentages represent evidence-derived exploratory weights and should not be interpreted as validated predictive coefficients or definitive clinical decision thresholds.

In the five-domain model, the resulting domain-specific scores were 52.7 for the clinical domain, 69.4 for the metabolic/endocrine domain, 68.2 for the imaging domain, 49.9 for the psychological domain, and 53.7 for the inflammatory domain. Application of the provisional multidomain equation yielded a total FS burden score of 59.8/100. When expressed as relative weighted contributions within this example, the profile was approximately 27.9% metabolic/endocrine, 26.1% imaging, 18.6% clinical, 16.7% psychological, and 10.8% inflammatory. These distributions represent conceptual approximations derived from the current literature and should be interpreted as illustrative research-use outputs. To provide an integrated visual synthesis of the evidence-derived framework, Figure 3 summarises the five multidomain contributors retained in the model, their provisional relative contribution, and the intended research-use interpretation of the framework.

## 4. Discussion

The present study developed an evidence-derived multidomain conceptual framework for FS and translated it into a research-use operational prototype for patient-level phenotyping. The main finding is that FS is not adequately represented by clinical presentation alone. Current evidence supports a multidomain structure in which clinical, metabolic/endocrine, imaging, psychological, and inflammatory contributors are all relevant, but with different degrees of evidential maturity and different levels of provisional weight. This interpretation is aligned with recent integrative research framing FS as a condition shaped by the interaction of local tissue pathology with metabolic, inflammatory, pain-processing, behavioural, and psychosocial mechanisms [4,9,15].

This framework extends previous multidomain and biopsychosocial interpretations of FS. Existing models have been valuable in moving the field away from a purely local capsular view of the condition, but they have generally remained descriptive and have not provided a transparent structure for organising different contributors into a patient-level profile. The present framework addresses this gap by separating two linked phases: first, systematic identification and synthesis of evidence across clinically meaningful domains; and second, exploratory framework development and operationalisation. In this sense, the model should be interpreted as a structured conceptual and research-use framework rather than as a validated clinical prediction tool.

The prominence of the metabolic/endocrine sphere is clinically relevant. DM-related burden and glycaemic control emerged as the strongest internal components of this sphere, consistent with evidence showing that FS is more frequent in people with DM and may follow a more severe or prolonged course [9]. Other endocrine–metabolic factors, including obesity, thyroid dysfunction, altered lipid metabolism, and liver-associated metabolic markers, contributed additional but smaller layers of information [7,8,9,10]. These variables may define a systemic biological context in which advanced glycation, chronic low-grade inflammation, oxidative stress, endocrine dysfunction, and pro-fibrotic signalling influence capsular fibrosis and recovery potential [4,14,15,87]. This supports the relevance of metabolic profiling as part of FS phenotyping rather than as a secondary comorbidity screen alone.

The imaging sphere also emerged as a major component of the framework. MRI and ultrasound findings were integrated within a single imaging domain because the retained evidence converged on related structural and activity-related tissue features rather than on clearly independent modality-specific constructs. Capsular structural biomarkers, edoema- or hyperintensity-related activity signs, vascular imaging features, and compensatory sonographic findings may provide information about tissue phenotype, disease phase, and prognosis. However, these findings should not be interpreted as direct equivalents of clinical severity. Instead, imaging appears to provide a complementary tissue-level layer that may help explain why patients with similar symptoms can differ in structural or activity-related disease expression.

The psychological sphere underwent important refinement during framework development. Earlier versions of the model captured distress/mood and psychosocial quality of life, but targeted inclusion of sleep-focused research showed that sleep-related burden was too relevant to omit. At the same time, current evidence did not support sleep as a fully independent sixth sphere because sleep disturbance overlaps substantially with distress, pain-related burden, irritability, and psychosocial functioning [21,80]. Therefore, sleep-related burden was nested within the psychological sphere while retaining its status as a major subdomain. This structure recognises that the psychological contribution to FS extends beyond anxiety and depression and includes distress/mood, sleep-related burden, psychosocial quality of life, and psychological interference with perceived recovery [19,20,21,88,89,90,91]. Future studies should determine whether sleep adds independent explanatory value beyond the broader psychological domain, ideally using longitudinal designs, validated sleep instruments, and, where feasible, objective sleep measures.

The inflammatory sphere remains the least secure domain in the framework and should be interpreted with particular caution. This does not imply that inflammation is biologically unimportant in FS. Mechanistic and translational research supports a role for inflammatory activation, cytokine imbalance, matrix remodelling, and failed fibrosis resolution [42]. In the retained case–control study, patients with FS showed higher serum IL-1β levels than controls, and an IL-1β polymorphism was associated with susceptibility, whereas MMP-3, TGF-β1, and GDF5 were not significantly associated within the same cohort. This supports retention of inflammation as a distinct domain, but also indicates that the direct retained signal is narrow and centred mainly on IL-1β-related biomarker and susceptibility evidence. Broader inflammatory-fibrotic mechanisms—including IL-10- and IL-17A-related pathways and repair/resolution cell populations—provide important contextual support but were not part of the retained quantitative evidence base used for weighting. Therefore, the lower weighting assigned to the inflammatory sphere reflects the limited maturity of the retained quantitative evidence rather than a judgement that inflammation is biologically unimportant [4,19,21,87,92].

Another implication of the framework concerns pain processing. Recent integrative research suggests that FS pain is not always reducible to local nociceptive input alone, but may involve interactions between peripheral inflammation, autonomic dysfunction, central and peripheral sensitisation, sleep disruption, and psychological amplification [93]. This view is consistent with maintaining clinical, psychological, and inflammatory spheres as analytically distinct components of the framework. However, analytical separation should not be interpreted clinically as evidence that these domains operate as independent compartments. Metabolic dysfunction, inflammatory activity, pain sensitisation, sleep disturbance, psychological distress, physical inactivity, and movement avoidance are likely to interact dynamically within individual patients. Accordingly, the framework should be interpreted as a tool for clinical orientation and research prioritisation rather than as a rigid phenotypic categorisation system [23].

A strength of the present study is that it moves beyond conceptual description and into operationalisation. The prototype demonstrates that the multidomain framework can be translated into measurable patient-level inputs and can generate both a total multidomain burden score and a sphere-specific contribution profile. Before validation, however, clinicians should not interpret these scores as diagnostic thresholds, prognostic probabilities, or treatment-allocation rules. The sphere-specific outputs are best understood as structured descriptive profiles that may help organise clinical reasoning and research stratification. The worked example was therefore included only to illustrate how the model can be operationalised; the value of 59.8/100 should not be interpreted as having established clinical meaning.

This distinction is particularly important for future clinical implementation. Some inputs included in the complete framework, especially inflammatory biomarkers and advanced imaging variables, are not routinely available in many rehabilitation settings. Therefore, the full prototype may be most appropriate at this stage for research cohorts or specialist settings. Future work may need to develop simplified versions based on routinely obtainable variables, while preserving the multidomain logic of the framework. Such simplified models should be derived and validated prospectively rather than assumed from the present exploratory structure.

Several limitations must be considered. First, the weighting procedure was evidence-derived and structured, but it was not estimated from individual-patient data, a single empirical prediction model, or an empirically estimated treatment-response model. Although explicit domain definitions, predefined assignment rules, independent reviewer procedures, risk-of-bias appraisal, and supplementary weighting tables improve transparency, traceability, and internal consistency, they do not eliminate investigator judgement. Another independent reviewer group could reasonably assign different weights when interpreting the same heterogeneous evidence base, particularly in domains supported by sparse, indirect, or methodologically variable data. Accordingly, the present weighting structure should be interpreted as an auditable first-step synthesis rather than as a fully reproducible or empirically derived coefficient system. Future iterations should complement structured reviewer judgement with formal Delphi or expert-consensus procedures, independent external replication of the weighting matrix, prospective individual-patient-data validation, and empirical derivation or recalibration in multicentre cohorts. Second, the search strategy may have introduced selection bias. The review was limited to three databases, English-language publications, open-access full texts, human adult studies, and studies published within the previous ten years. EMBASE was not searched. These criteria improved feasibility and ensured that all retained studies could be fully inspected and extracted, but they may have excluded relevant high-quality evidence published before the selected time window, in other languages, behind subscription barriers, or in additional databases. Because the present work was intended as a first exploratory framework rather than a definitive evidence map, the search was not repeated during revision. Future updates should remove the open access restriction, include additional databases such as EMBASE, consider broader language and date coverage where feasible, and test whether the resulting evidence base modifies the relative weighting of the domains.

Third, the retained evidence was methodologically heterogeneous. Risk-of-bias assessment showed recurring concerns regarding confounding, selection bias, outcome measurement, statistical adjustment, and reporting. These concerns were not evenly distributed across domains. The clinical sphere was the largest but also the most heterogeneous; imaging and biomarker studies provided biologically relevant signals but were constrained by protocol variability and limited longitudinal follow-up; psychological studies relied substantially on self-reported constructs that may overlap with pain, disability, and sleep-related burden; and the inflammatory sphere was supported only by limited direct observational evidence. Accordingly, the apparent balance of the framework should be interpreted in light of differences in both the quantity and confidence of evidence across domains.

Fourth, several assumptions were required to operationalise the prototype. Standardising all variables onto a 0–100 metric improves interpretability, but it may oversimplify nonlinear relationships or threshold effects for variables such as range-of-motion deficit, HbA1c, psychological distress, sleep disturbance, and imaging abnormalities. Similarly, proportional redistribution of weights in the presence of missing variables is pragmatic, but its impact on score stability has not yet been fully evaluated. The four-domain sensitivity specification excluding the inflammatory sphere suggested that the global worked-example score remained broadly stable, but this should be interpreted only as a preliminary robustness check, not as formal validation.

These limitations define the next phase of research. Future work should move beyond structured evidence-derived weighting toward independent replication and empirical refinement. Formal Delphi consensus procedures, external expert reassessment of the weighting matrix, prospective individual-patient-data validation, and statistical recalibration in multicentre cohorts will be necessary to determine whether the proposed domain weights are stable, reproducible, and clinically useful. Prospective validation in real FS cohorts is also necessary to assess the calibration, stability, feasibility, and clinical usefulness of the framework. Future studies should test alternative weighting scenarios, evaluate nonlinear and threshold-based scoring functions, quantify the impact of missing data, and determine whether sphere-specific profiles are associated with treatment response, recurrence, symptom persistence, and patient-centred outcomes [94]. Particular attention should be paid to whether sleep-related burden should remain nested within the psychological sphere or be modelled independently, and whether inflammatory biomarkers add stable phenotypic or prognostic value beyond clinical, metabolic/endocrine, imaging, and psychological data.

Finally, FS may represent a clinically meaningful opportunity for broader lifestyle-oriented intervention. Many patients present with sleep disturbance, reduced physical activity, metabolic dysfunction, stress-related burden, and altered recovery capacity. In this context, the shoulder complaint itself may function as an entry point for integrated health optimisation extending beyond local tissue management alone. The present framework does not prescribe treatment allocation, but it supports the rationale for future studies evaluating whether multidomain and lifestyle-informed rehabilitation strategies improve outcomes compared with approaches centred mainly on local shoulder impairment.

## 5. Conclusions

FS is more appropriately understood as a multidomain condition than as a purely local stiffness syndrome. In the present evidence-derived framework, clinical presentation, metabolic/endocrine burden, imaging phenotype, psychological factors, sleep-related burden, lifestyle-related contributors, and inflammatory biology were organised into an integrated whole-person model. Metabolic/endocrine, imaging, and clinical factors emerged as major contributors, psychological factors added a substantial complementary layer, and inflammatory biology remained biologically relevant but quantitatively more provisional.

This study provides a first conceptual and operational platform for multidomain phenotyping in FS. The framework is exploratory and should not be interpreted as a validated clinical decision-support tool, a definitive disease-partitioning system, or a treatment-allocation algorithm. Instead, it offers a structured basis for future validation, recalibration, and refinement of patient-level phenotyping strategies. Future research should determine whether these interacting domains can improve prognostic stratification, guide comprehensive rehabilitation planning, and support more personalised but integrated management of FS.

## Figures and Tables

**Table 1:** Detailed search strategies.

Database	Search Strategy	Filters
PubMed	((“adhesive capsulitis”[Title/Abstract] OR “frozen shoulder”[Title/Abstract]) AND (cohort[Title/Abstract] OR longitudinal[Title/Abstract] OR “follow-up”[Title/Abstract] OR prospective[Title/Abstract] OR retrospective[Title/Abstract])) AND (SPADI[Title/Abstract] OR “Shoulder Pain and Disability Index”[Title/Abstract] OR VAS[Title/Abstract] OR “visual analog*”[Title/Abstract] OR DASH[Title/Abstract] OR “range of motion”[Title/Abstract] OR ROM[Title/Abstract] OR “external rotation”[Title/Abstract] OR flexion[Title/Abstract] OR abduction[Title/Abstract])	Humans; Adults; English Language; Free full text; last 10 years
Web of Science	((TS = (“adhesive capsulitis” OR “frozen shoulder”)) AND TS = (cohort OR longitudinal OR “follow-up” OR prospective OR retrospective)) AND TS = (SPADI OR VAS OR DASH OR “range of motion” OR ROM)	Document Types = Article; English language; Open access; last 10 years
ScienceDirect	Title, abstract or author-specified keywords (“adhesive capsulitis” OR “frozen shoulder”) (cohort OR longitudinal OR prospective OR retrospective) (SPADI OR VAS OR DASH OR “range of motion” OR ROM)	Article Types = research articles; English language; Open access; last 10 years

**Table 2:** Characteristics of the included studies.

Study	Study Design	Population	Sample Size	Groups	Intervention Type	Intervention Details	Follow-Up	Outcomes
Lychagin et al. 2022 [25]	Prospective cohort comparative study	Patients aged 47–60 years with FS; disease duration 3 months–5 years; stage I or II FS.	n = 42	AC1/stage I = 24; AC2/stage II = 18; each stage allocated to PRP or PN subgroup.	Intra-articular biological injection therapy	PRP vs. PN intra-articular ultrasound-guided injections.	1 week, 1 month, 3 months	Pain VAS, DASH, SST, shoulder ROM, quality of life/function
Jain et al. 2023 [26]	Prospective single-blind observational comparative study	Adults with idiopathic FS (stage I–II), shoulder pain > 3 months	n = 65	SSNB group (n = 32) vs. HD group (n = 33)	Ultrasound-guided suprascapular nerve block vs. HD	SSNB: triamcinolone + bupivacaine injected into suprascapular notch under US guidance. HD: fluoroscopy-guided capsular distension with saline, contrast, lignocaine, triamcinolone, and bupivacaine	2, 6, 12, and 24 weeks	SPADI (pain/disability), active ROM (abduction, flexion, internal rotation), pain during procedure, functional recovery
Barman et al. 2021 [27]	Prospective observational cohort study	Patients with diabetes mellitus and FS for <6 months	n = 70	PRP group: n = 35; PT group: n = 35	PRP vs. PT	PRP: single 4 mL ultrasound-guided intra-articular PRP injection into the glenohumeral joint. PT: 10 sessions over 2 weeks including TENS, ultrasound therapy, and passive joint mobilisation; both groups received home exercise advice	3, 6, and 12 weeks	VAS pain, SPADI, active and passive ROM, acetaminophen use, adverse events
Lee et al. 2022 [28]	Comparative observational study: prospective breast cancer surgery cohort + retrospective idiopathic AC control group	Patients with FS after breast cancer surgery and patients with idiopathic adhesive capsulitis	n = 67	BC surgery group: n = 23; idiopathic FS control group: n = 44	Hydrodilatation with corticosteroid injection	Ultrasound-guided intra-articular hydrodilatation using 50 mL injectate: triamcinolone, lidocaine, and saline; both groups received home exercise education	BC group assessed at baseline, 2 weeks, and 4 weeks; CON group assessed at baseline for biomechanical comparison	Shoulder ROM, SPADI pain and disability scores, capsular capacity, maximal pressure, capsular stiffness
Song et al. 2021 [29]	Retrospective cohort study with propensity score matching	Patients with primary FS refractory to ≥1 month of conservative treatment	n = 141	MUA only: n = 60; MUA + ISI: n = 81. After matching: 44 per group	Manipulation under anaesthesia with or without intra-articular steroid injection	MUA performed after cervical nerve root block. ISI group received triamcinolone acetonide + lidocaine immediately after MUA. Repeat MUA was offered at 1 week if response was insufficient	1, 2, and 4 weeks; phone follow-up at 3 and 6 months	SPADI pain, disability and total score; passive ROM; global impression of change; need for additional treatment; adverse events
Li et al. 2022 [30]	Comparative observational study: prospective breast cancer surgery cohort + retrospective idiopathic AC control group	Patients with FS after bc surgery and patients with idiopathic FS.	n = 67	BC surgery group: n = 23; idiopathic FS control group: n = 44	Hydrodilatation with corticosteroid injection	Ultrasound-guided intra-articular hydrodilatation using 50 mL injectate: triamcinolone, lidocaine, and saline; both groups received home exercise education	BC group assessed at baseline, 2 weeks, and 4 weeks; CON group assessed at baseline for biomechanical comparison	Shoulder ROM, SPADI pain and disability scores, capsular capacity, maximal pressure, capsular stiffness
Inglese et al. 2024 [31]	Retrospective cohort study	Patients < 55 years with phase III FS and severe ROM limitation	n = 110	Single treatment cohort	Awake shoulder manipulation under brachial plexus block	Ultrasound-guided interscalene brachial plexus block followed by standardised passive shoulder manipulation and complementary rehabilitation programme	4 months and 1 year eligibility follow-up	NPRS pain, Simple Shoulder Test, shoulder ROM, patient satisfaction, complications
Yildiz et al. 2018 [32]	Retrospective comparative cohort study	Retrospective comparative cohort study	n = 72	Group I: no concomitant intra-articular pathology, n = 46; Group II: concomitant intra-articular lesions, n = 262	Arthroscopic capsular release	Arthroscopic 360° capsular release. Concomitant non-repaired lesions included SLAP lesions, partial rotator cuff tears, impingement; some received debridement, biceps tenotomy, subacromial decompression or acromioplasty as appropriate	Minimum 12 months; mean 26 months in Group I and 15 months in Group II	ROM, Constant score, VAS pain, complications
Martens et al. 2024 [33]	Retrospective cohort study	Patients with refractory FS for >6 months, unresponsive to conventional treatment	n = 32	Single treatment cohort	Continuous SSNB + intensive multidisciplinary rehabilitation	Ultrasound-guided continuous suprascapular nerve blockade with ropivacaine for 10 days, combined with daily physiotherapy and occupational therapy during hospitalisation, followed by outpatient rehabilitation 3 times/week	Baseline, days 3, 6, 10, 30, 90, and 180	Active and passive ROM, VAS pain, DASH score, adverse events
Bongiorno et al. 2023 [34]	Case report	51-year-old woman with diabetes and adhesive capsulitis of the left shoulder for ~6 months; steroid injection contraindicated	1	Single patient	Pulsed radiofrequency of the suprascapular nerve	Single ultrasound-guided pulsed radiofrequency treatment of the left suprascapular nerve; no physiotherapy during the first 3 weeks	3 weeks; tele-visit at 12 weeks	NRS pain, SPADI, ROM, kinematic analysis, Jerk index, surface electromyography
Zhou & Cheng 2025 [35]	Retrospective comparative cohort study	Patients with primary adhesive capsulitis of the shoulder; symptoms ≥ 3 months; exclusion of secondary causes and major structural pathology	n = 72	MUA group: 36; Conservative treatment group: 36	Manipulation under anaesthesia vs. conservative treatment	MUA under ultrasound-guided interscalene brachial plexus block followed by structured 3-month rehabilitation; control group received standardised conservative treatment	1, 3, 6, and 12 months	ROM, Constant–Murley score, VAS pain, patient satisfaction, return-to-work time, complications
Vastamäki et al. 2016 [36]	Retrospective cohort study	Patients with idiopathic FS, comparing those with and without diabetes	n = 178	Diabetes: n = 27, without diabetes: n = 151	Conservative treatment or manipulation under anaesthesia	88 shoulders received conservative treatment/observation and 110 underwent MUA	Mean 9.7 years	ROM, pain VAS, Constan–-Murley score, Simple Shoulder Test, comparison with contralateral shoulder, insulin dependency
Liu et al. 2025 [37]	Prospective cohort study	Patients with primary unilateral FS refractory to ≥3 months of conservative treatment	n = 156	FS stages by symptom duration: Stage 2: 95, Stage 3: 31, Stage 4: 30	Manipulation under anaesthesia with objective force measurement	MUA performed after failed nonsurgical treatment; releasing force measured using handheld dynamometer during forward flexion, external rotation and internal rotation	1, 3 and 6 months	Releasing force, tear value, peak value, ROM, VAS, Oxford Shoulder Score
Pasqualini et al. 2024 [38]	Prospective case series	Patients with idiopathic FS refractory to physiotherapy and corticosteroid injection	n = 73	Single group	Arthroscopic capsular release	Anteroinferior arthroscopic capsular release with postoperative rehabilitation	1, 2, 4, 6 and 12 months	VAS, ASES, SANE, Constant score, MCID, PASS, ROM
Luo et al. 2025 [39]	Retrospective comparative study	Patients with idiopathic FS undergoing arthroscopic release	n = 73	LHBT tenotomy: n = 41; LHBT left in situ: n = 32	Arthroscopic capsular release with or without long head of biceps tendon tenotomy	All underwent capsular release, coracohumeral ligament release and subacromial decompression; tenotomy performed when LHBT inflammation was present	1, 3, 6, 12, 24 months and final follow-up	Both groups improved long term. LHBT tenotomy showed better early pain reduction at 1 and 3 months and better external rotation from 1 to 12 months. No significant final follow-up difference between groups.
Kumar et al. 2017 [40]	Retrospective comparative study	Female patients aged 40–60 years with idiopathic FS treated with physiotherapy	n = 41	PT only: 20; PT + injection: 21	Physiotherapy ± intra-articular corticosteroid injection	PT programme alone versus single intra-articular injection of 40 mg methylprednisolone followed by physiotherapy	12 weeks	VAS pain score and shoulder ROM: flexion, abduction, internal rotation, external rotation
Dakkak et al. 2024 [41]	Retrospective cohort study	Patients aged 30–75 years with FS, included diabetic and non-diabetic patients	n = 150	Diabetic: n = 25; non-diabetic: n = 125	Multimodal non-surgical intervention	Ultrasound-guided suprascapular nerve block + ultrasound-guided glenohumeral hydrodilatation with corticosteroid/local anaesthetic + immediate manual physical therapy, followed by PT	3 months	VAS pain score, active forward flexion ROM, active external rotation ROM, safety/complications
Mertens et al. 2022 [42]	Longitudinal multicentre observational study	Patients with frozen shoulder stage 1 or 2	n = 49	Single cohort	Prognostic/clinical profile assessment	ROM limitation, diabetes mellitus, thyroid disorder, autonomic symptoms, pain sensitivity/central pain processing	9 months	SPADI, SF-36 quality of life, ROM, quantitative sensory testing
Lesevic et al. 2021 [43]	Case–control/retrospective cohort	Idiopathic FS treated with fluoroscopic glenohumeral corticosteroid injection	n = 728	Patients requiring vs. not requiring LOA/MUA or repeat injection	Fluoroscopic intra-articular corticosteroid + anaesthetic injection	Immediate VAS pain reduction after injection, pre/post-injection VAS, need for LOA/MUA, repeat injection	≥1 year	Immediate pain relief did not predict MUA or repeat injection; MUA rate was low: 5.1%
Li et al. 2024 [44]	Retrospective comparative study	Idiopathic unilateral FS refractory to conservative treatment	n = 80	Multisite injection vs. arthroscopic capsular release	MI: lidocaine + triamcinolone at biceps long head, posteroinferior capsule, coracohumeral ligament ± trigger points; ACR: arthroscopic capsular release	VAS, ROM, OSS, DASH, diabetes subgroup, complications	1, 3, 6 months	Both treatments significantly improved pain, ROM and function. ACR had better IR/ER at 1 month, but no differences at 6 months. Diabetes did not worsen outcomes. MI was considered simpler, safe, lower-cost, and a potential first option before ACR.
Kim et al. 2021 [45]	Retrospective study	Patients with bilateral primary FS	n = 165	Injected shoulder vs. non-injected shoulder (within-patient comparison)	Unilateral ultrasound-guided intra-articular corticosteroid injection (triamcinolone acetonide + lidocaine) in the more painful shoulder	Pain (NRS), passive ROM (abduction, external rotation, flexion, hyperextension, internal rotation), diabetes subgroup analysis	Mean 6.7 weeks	Significant improvement in pain and passive ROM in both injected and non-injected shoulders, although greater improvement in the injected shoulder. Response was less pronounced in diabetic patients. Authors suggest initial unilateral injection with observation rather than simultaneous bilateral injections.
Mardani-Kivi et al. 2021 [46]	Retrospective study	Patients with unilateral FS refractory to ≥6 months of conservative treatment	n = 51	Aetiology subgroups: idiopathic/post-surgical/post-traumatic	Arthroscopic capsular release (global release + postoperative physiotherapy)	Pain (VAS), shoulder function (Constant Score), satisfaction (Simple Shoulder Test), ROM (forward flexion, abduction, internal/external rotation), effect of age, sex, diabetes, aetiology	Mean 49.3 months (range 2–6 years)	Significant improvement in pain, function, satisfaction, and ROM at 6 months and maintained at long-term follow-up. Age, sex, and diabetes did not significantly affect outcomes. Patients with post-surgical adhesive capsulitis had consistently worse recovery than idiopathic or post-traumatic cases. Arthroscopic release was effective with very low complication rates.
Mert et al. 2025 [47]	Retrospective single-centre comparative study	Patients with primary FS refractory to conservative treatment	n = 54	MUA under general anaesthesia/MUA under ultrasound-guided neuronal block/control exercise group	Manipulation under anaesthesia vs. ultrasound-guided interscalene neuronal block vs. self-directed rehabilitation	ROM: abduction, flexion, external rotation; pain by VAS; complications	Immediate and 1 week after intervention; control at 4 and 6 weeks	Both intervention groups improved significantly versus baseline and control. General anaesthesia showed better immediate flexion improvement, while ultrasound-guided neuronal block showed better abduction, external rotation, and pain relief at 1 week. No major complications were reported.
Xu et al. 2024 [48]	Retrospective observational study	Patients with FS in painful/freezing stage	n = 198	Celecoxib/transdermal buprenorphine patch/buprenorphine patch + celecoxib	Pharmacological analgesic treatment	Pain by VAS at rest and movement; shoulder function by Constant–Murley Score; satisfaction; adverse reactions	1, 4, 8 and 12 weeks	All groups improved, but the combined buprenorphine patch + celecoxib group had the lowest VAS scores, highest CMS scores, and greatest satisfaction. Adverse reactions did not differ significantly between groups.
Albana et al. 2022 [49]	Retrospective comparative study	Patients aged 40–60 years with FS	n = 31	Hydrodilatation alone/hydrodilatation + suprascapular nerve block	Hydrodilatation with corticosteroid, with or without SSNB	Pain by VAS; and DASH	During intervention, 1 month and 6 months	Adding SSNB reduced pain during the procedure and at 1 month, and improved short-term function. At 6 months, differences between groups were no longer significant.
Menekse et al. 2024 [50]	Retrospective observational study	Patients with frozen shoulder	n = 50	MUA alone/MUA + open bursectomy + biceps tendon capsule release	Surgical treatment comparison	Pain by VAS; ROM; quality of life by SPADI	Postoperative follow-up, duration not clearly specified	Both groups improved, but the combined approach showed greater pain reduction, better ROM and better SPADI scores than closed manipulation alone.
Sıvacıoğlu et al. 2025 [51]	Retrospective analysis	Patients with FS and concomitant rotator cuff tear	n = 29	Single group	Simultaneous arthroscopic global capsular release + rotator cuff repair	ROM; VAS pain; Constant score; complications; re-tear	Mean 14 months	Significant improvement in ROM, pain and function. VAS decreased from 7.1 to 1.2 and Constant score improved from 37.5 to 72.3. No re-tears were reported; 2 diabetic patients had persistent ROM limitation.
Haider et al. 2022 [52]	Prospective cohort study	Patients with FS	n = 305	Single group	Platelet-rich plasma injection	Pain by VAS; percentage pain reduction; adverse effects	6 weeks	VAS improved from 6.56 to 2.42. Pain reduction was 64.6% on average; 87.5% achieved ≥50% pain improvement. No complications were reported.
Jung et al. 2019 [53]	Retrospective cohort study	Patients with FS	n = 102	SSNB + IAI vs. IAI alone	Suprascapular nerve block plus intra-articular corticosteroid injection vs. corticosteroid injection alone	ROM, pain/function VAS, ASES, KSS, Constant, SST, SPADI	2 weeks, 2 months, minimum 1 year	Both groups improved, but SSNB + IAI showed greater improvement in function, ASES, SST, SPADI, forward flexion and abduction at 2 months. At ≥1 year, FVAS and ASES remained better in the combined group.
Atici et al. 2021 [54]	Retrospective cohort study	Patients with FS	n = 18	Single treatment cohort	High-dose oral prednisolone, starting at 1 mg/kg/day with gradual tapering	ROM, VAS pain, DASH, Constant–Murley, ASES, adverse effects	4 weeks and 6 months.	Rapid improvement in shoulder motion and pain at 4 weeks, maintained at 6 months. DASH and ASES improved significantly later, at 6 months. No major adverse effects or need for surgery were reported.
Saito et al. 2021 [55]	Retrospective cohort study	Patients with FS treated with MUC	n = 70	Good clinical result: ASES ≥80; poor clinical result: ASES <80	Shoulder manipulation under ultrasound-guided cervical nerve root block	Outpatient MUC under C5–C6 cervical nerve root block; patients had failed ≥3 months of conservative treatment	1 year	Pain, ROM and ASES improved significantly. Diabetes mellitus was the only independent negative prognostic factor for poor outcome after MUC: OR 51.0, 95% CI 10.9–237, *p* = 0.01.
Saito et al. 2023 [56]	Retrospective cohort study	Patients with FS treated with manipulation under ultrasound-guided cervical nerve root block	n = 70	Good result: ASES ≥ 80; Poor result: ASES < 80	Manipulation under ultrasound-guided cervical nerve root block; prognostic factor analysis	Diabetes mellitus, age, sex, symptom duration, baseline pain, ROM, ASES score	1 year	Pain, ROM and ASES improved significantly after treatment. Diabetes mellitus was the only independent risk factor for poor outcome, with OR 51.0.
Wang et al. 2025 [57]	Retrospective cohort study	Patients with FS	n = 130	Single cohort	Two ultrasound-guided intra-articular corticosteroid injections, 6 weeks apart	Pain, forward elevation, external rotation, internal rotation	Baseline, 6 weeks, 12 weeks	Pain, forward elevation and internal rotation improved after the first injection. External rotation improved significantly only after the second injection.
Satora et al. 2021 [58]	Retrospective comparative study	Patients with FS ≤ 6 months	n = 59	Surgical group n = 30; nonsurgical group n = 29	Arthroscopic capsular release + corticosteroid injection + physiotherapy vs. corticosteroid injection + physiotherapy	ROM, pain, DASH function	3, 6 and 12 months	Surgery produced faster improvement in ROM and function at 3 and 6 months. At 12 months, both groups had similar clinical results. Pain improved similarly in both groups.
Shang et al. 2025 [59]	Multicenter retrospective study	Patients with FS	n = 258	HAG n = 123; HG n = 135	Ultrasound-guided glenohumeral hydrodilatation + acupotomy release vs. hydrodilatation alone	PROM, Constant–Murley Score, VAS, SPADI, adverse events	3, 6 and 12 weeks	Both groups improved, but combined hydrodilatation + acupotomy showed better mid-term ROM, function and SPADI outcomes. Pain improvement was superior at 3 and 6 weeks, but similar by 12 weeks. No serious adverse events were reported.
Yuan et al. 2018 [60]	Retrospective study	Patients with FS	n = 134	Single Cohort	Two ultrasound-guided intra-articular corticosteroid injections, 6 weeks apart	Pain, forward elevation, external rotation, internal rotation	Baseline, 6 weeks, 12 weeks	Pain, forward elevation and internal rotation improved after the first injection. External rotation improved significantly only after the second injection.
Bai et al. 2023 [61]	Retrospective study	Middle-aged and older patients with FS after arthroscopic capsular release	n = 85	TXA n = 28; cocktail n = 26; cocktail + TXA n = 31	Postoperative intra-articular infusion of TXA, analgesic cocktail, or cocktail + TXA	Drainage, hospital stay, pain, Neer score, ASES, ROM, complications	1 day, 1 week, 1 month, 3 months	Cocktail + TXA reduced pain and bleeding, shortened early recovery, and produced better early shoulder function than TXA alone or cocktail alone. All groups improved by 3 months, but recovery was greatest with cocktail + TXA.
Kim et al. 2019 [62]	Retrospective case-controlled comparative study	Patients with idiopathic FS treated with hydraulic distension	n = 47	Pumping n = 24; non-pumping n = 23	US-guided capsule-preserving hydraulic distension, with or without “pumping technique”	ROM, VAS, SPADI, complications	6 months	Both groups improved pain, ROM and SPADI after the second injection. Pumping produced better ROM and SPADI disability improvement, but pain reduction was similar. No serious complications.
Wu et al. 2025 [63]	Prospective cohort study	Patients with FS	n = 60	PRF n = 30; nerve block n = 30	Combined suprascapular and axillary nerve pulsed radiofrequency vs. nerve block	NRS pain, SPADI, PROM, adverse events	6 months	Both treatments improved pain, function and PROM. PRF produced greater improvement in activity/night pain, SPADI and most ROM measures at 3–6 months. No serious complications.
Hyun Kim et al. 2020 [64]	Retrospective diagnostic accuracy study	Patients with FS vs. healthy controls		n = 74 (39 FS, 35 controls)	SMI vs. conventional US and PDUS	SMI vascular area, PDUS vascular area, CHL thickness, rotator interval echogenicity, ROM, pain, symptom duration	Cross-sectional diagnostic assessment	SMI vascular area was higher in adhesive capsulitis and had the best diagnostic performance (AUC 0.90). SMI was superior to PDUS for detecting vascular flow. Greater SMI vascular flow correlated with worse external rotation and forward flexion.
Hwan Kim et a. 2018 [65]	Diagnostic correlation study	Patients with FS	n = 44	Affected shoulder vs. unaffected shoulder	Ultrasound measurement of axillary recess capsule thickness, compared with MRI	Axillary recess capsule thickness, ROM limitation, VAS, ASES, SSV	Cross-sectional imaging assessment	Ultrasound showed greater capsule thickness in affected shoulders than unaffected shoulders: 4.4 vs. 2.2 mm. Ultrasound thickness strongly correlated with MRI thickness, r = 0.83. A cutoff of 3.2 mm showed good diagnostic accuracy. Capsule thickness did not correlate with specific ROM limitation patterns.
Stella et al. 2022 [66]	Cross-sectional diagnostic study	Adults with shoulder pain and stiffness evaluated for FS.	n = 1486	FS patients vs. healthy controls; affected vs. contralateral shoulder	Ultrasound diagnostic assessment	Axillary pouch, CHL/SGHL thickness, LHBT sheath effusion, infraspinatus tendon sliding during passive external rotation	Cross-sectional	Typical US findings were axillary pouch thickening in 100%, LHBT sheath effusion in 71%, CHL/SGHL thickening in 88%, and reduced infraspinatus sliding in 73%. AP thickening had high diagnostic accuracy, supporting ultrasound as useful to confirm clinical FS.
Park et al. 2016 [67]	Retrospective imaging-clinical correlation study	Patients with FS assessed by shoulder MRI	n = 103	Clinical stages 1–4	Fat-suppressed T2-weighted MRI evaluation	Axillary recess capsule edoema/thickness, extracapsular edoema, subcoracoid fat obliteration, biceps sheath effusion, pain, ROM, clinical stage	Cross-sectional MRI-clinical assessment	Anterior extracapsular edoema correlated with limitation in external rotation and abduction. Humeral axillary recess edoema correlated with external rotation limitation and was more common in early stages. Humeral capsule thickness correlated with pain and was greater in stage 1. MRI may help assess clinical impairment and disease stage.
Tang et al. 2024 [68]	Retrospective study	Patients with unilateral FS	n = 362	4 groups: no cervical muscle involvement, scalene involvement, levator scapulae involvement, both muscles involved	Sonographic evaluation of cervical muscle involvement	Shoulder ROM, scalene complex and levator scapulae thickening/hypoechoic changes, clinical risk factors	Retrospective clinical record review	Patients with scalene or levator scapulae involvement had significantly greater shoulder flexion, abduction, external rotation, and total ROM than those without involvement. Ultrasound showed thickening and hypoechoic changes in these muscles. Authors suggest these muscles may compensate for restricted shoulder motion.
Liang et al. 2025 [69]	Retrospective study	Patients with FS to conservative treatment	n = 25	Single group	Transarterial embolization using imipenem/cilastatin	Pain, DASH, ROM, MRI inflammation findings	1, 3, and 6 months; MRI at 3 months	TAE significantly reduced pain, improved Quick DASH and ROM, and decreased MRI signs of inflammation in the axillary recess and rotator interval. Clinical success was 88%, with no severe adverse events.
Guillet et al. 2021 [70]	Prospective monocentric study	Adults with clinically diagnosed FS confirmed by MRI	n = 132	No intervention groups; observational comparison based on MRI findings (high vs. low IGHL signal intensity, ligament thickness categories)	Diagnostic/prognostic imaging assessment	Shoulder MRI evaluation assessing IGHL signal intensity and thickness, coracohumeral ligament thickness, correlated with Constant–Murley Score, pain duration, ROM, and symptom characteristics	Approximately 1 year (9–13 months in follow-up subgroup)	High IGHL T2 signal was associated with shorter pain duration, nocturnal pain, and lower mobility, suggesting early inflammatory disease stage. Greater IGHL thickness (>4 mm) was associated with better clinical improvement at follow-up, whereas thinner ligaments (≤3 mm) were associated with worse prognosis.
Choi & Kim 2020 [71]	Retrospective observational study	Patients with FS of the shoulder (symptom duration ≤ 9 months, restricted passive ROM, no other shoulder pathology)	n = 29	No intervention groups; correlation analysis between MRI findings and clinical features	Diagnostic imaging assessment	Standardised 3T shoulder MRI evaluating capsular thickness (humeral, glenoid, maximal axillary, anterior capsule), coracohumeral ligament thickness, capsular hyperintensity, rotator interval abnormalities, and obliteration of subcoracoid fat triangle; correlated with ROM, pain (VAS), and symptom duration	Cross-sectional (mean MRI performed 16 days after clinical assessment; no longitudinal follow-up)	Maximal axillary capsular thickness and humeral capsular thickness were associated with worse internal rotation. Capsular hyperintensity in the axillary recess correlated with reduced abduction and forward flexion. Humeral capsular hyperintensity correlated with shorter symptom duration (suggesting early inflammatory stage). No MRI findings correlated with pain severity. CHL thickening and fat triangle obliteration were diagnostically present but not linked to symptom severity.
Chen et al. 2017 [72]	Case–control genetic association study	Chinese Han patients with FS in the freezing stage, compared with healthy controls	n = 92 (42 FS, 50 controls)	PFS group vs. healthy control group	Observational biomarker/genetic study	Genotyping of SNP polymorphisms in IL-1β (rs1143627), MMP-3 (rs650108), TGF-β1 (rs1800469), and GDF5 (rs143383) using MassARRAY; serum IL-1β measured by ELISA	Cross-sectional (single assessment)	IL-1β rs1143627 CC genotype was associated with reduced risk of PFS compared with TT genotype. Serum IL-1β levels were significantly higher in PFS patients. No significant association was found for MMP-3, TGF-β1, or GDF5 polymorphisms. Findings support a possible inflammatory genetic susceptibility mechanism in PFS.
Hamed et al. 2024 [73]	Cross-sectional observational study	Adults with FS	n = 32	No treatment groups; gender-based subgroup analysis	Observational metabolic biomarker study	Blood biomarkers: AST, ALT, GGT, TSH, lipids, glucose-related markers, inflammatory markers, vitamin D, etc. Pain assessed with NRS; disability/function with SPADI	Single baseline assessment	Lower AST, ALT, GGT and TSH were associated with higher pain. TSH also correlated with worse SPADI. Regression showed GGT and TSH were the strongest predictors of pain. Suggests metabolic/liver–thyroid axis may contribute to pain and disability in frozen shoulder.
Takahashi et al. 2024 [74]	Retrospective case–control prognostic study	Patients with FS treated with MUC	n = 135 shoulders in 121 patients	Success group: 126 shoulders; recurrence group: 9 shoulders	Manipulation under ultrasound-guided cervical nerve root block	C5–C6 ultrasound-guided nerve root block with lidocaine, followed by shoulder manipulation and rehabilitation	3 months	Recurrence rate was 7.4%. Lower pre-MUC Constant Shoulder score was an independent risk factor for recurrence. Recurrence patients had lower ER, higher pain scores, and tended to have poorer glycaemic control.
Takahashi et al. 2025B [75]	Retrospective cohort study	Patients with FS treated with manipulation under ultrasound-guided cervical nerve root block	n = 126	Success group: 112 shoulders; refractory group: 14 shoulders	Manipulation under ultrasound-guided cervical nerve root block	C5–C6 nerve root block with lidocaine, followed by shoulder manipulation and post-procedure rehabilitation	12 months	ROM and functional scores improved in both groups. Refractory patients had worse outcomes at 12 months. Older age and diabetes mellitus were independent negative prognostic factors. Age cutoff for refractory outcome was 56 years.
Dimitri-Pinheiro et al. 2023 [76]	Prospective observational study	Patients FS	n = 202	Diabetic vs. nondiabetic patients	Ultrasound-guided hydrodistension	US-guided glenohumeral hydrodistension with 30–50 mL solution: saline, lidocaine, bupivacaine, and 40 mg triamcinolone; followed by immediate exercises	2 years	VAS and DASH improved significantly at 2 years. Recurrence occurred in 28/202 patients. Diabetes was significantly associated with higher recurrence and shorter time to recurrence. No adverse effects reported.
Dimitri-Pinheiro et al. 2022B [77]	Retrospective longitudinal observational study	Patients with FS with ultrasound-guided hydrodistension	n = 120	Patients with T2D vs. without diabetes	Ultrasound-guided hydrodistension	Injection under ultrasound guidance of corticosteroid + saline solution into the glenohumeral capsule	6–12 months	Baseline FS severity was similar between groups. Patients with T2D had more relapse/reintervention, worse post-treatment pain, and worse DASH score. No significant worsening in HbA1c, fasting glucose, weight, or lipid profile after treatment.
Mulligan et al. 2015 [78]	Cross-sectional epidemiological study	Adults with shoulder disorders	n = 343	SAIS, rotator cuff tear, glenohumeral osteoarthritis, FS	No intervention	Clinical assessment + questionnaires: PSQI, ASES, SANE, VAS pain	Single assessment	Sleep quality was poor across all shoulder disorders, but worst in FS, which had significantly poorer PSQI scores, especially sleep quality, duration, and habitual sleep efficiency. Pain scores were not significantly different between groups.
Khan et al. 2025 [79]	Analytical cross-sectional study	Patients with FS	n = 111	Sleep quality categories; irritability levels	No intervention	PSQI for sleep quality; DASH-based assessment for disability/irritability	Single assessment	71.2% had significant sleep disturbance and 25.2% severe sleep difficulty. Moderate irritability was present in 56.8%, high irritability in 29.7%. Frozen shoulder irritability showed a significant association with sleep disturbance.
Toprak & Erden 2019 [80]	Prospective case–control study	Patients with FS and healthy controls	n = 148	76 FS patients; 72 healthy controls	No intervention	Assessment with VAS, BAI, BDI, PSQI and WHOQoL-BREF	Single assessment	FS patients had higher pain and anxiety, poorer sleep quality, and lower physical, psychological and environmental QoL. Depression was not significantly different. Sleep disturbance and habitual sleep efficiency were significantly worse in FS.
Fonseca et al. 2025 [21]	Cross-sectional study	Individuals with FS	n = 96	Primary FS and secondary intrinsic FS	No intervention	Psychological factors and sleep quality assessed using HADS, PSEQ-10, TSK-11, PCS, PSQI; pain/disability assessed with SPADI	Baseline only	Pain self-efficacy, kinesiophobia and BMI explained 22.3% of disability variance. Pain self-efficacy and anxiety explained 21.2% of activity-related pain variance. Sleep quality, depression and catastrophizing were not significantly associated in final models.
Bhagade & Sreeraj 2018 [81]	Explorative cross-sectional correlation study	Patients with FS and sleep disturbance, without psychological problems	n = 60	Single FS group	No intervention	Assessment with SPADI, PSQI and SF-36	Single assessment.	Sleep disturbance showed moderate positive correlation with pain and disability, strong correlation with total SPADI, and negative correlations with several QoL domains, especially general health.
Tache-Codreanu et al. 2025 [82]	Retrospective observational study	Patients with FS treated with RSWT	n = 40	BMI groups: normal weight, overweight, obese	Radial shock wave therapy + conventional physiotherapy	10-day physiotherapy protocol plus 5 weekly RSWT sessions; outcomes measured with VAS, SPADI, ROM and PGIC	Immediate post-treatment and 1 month	Pain, disability and ROM improved significantly. Higher BMI correlated with greater improvements in SPADI, VAS, extension and internal rotation. Most changes exceeded MCID thresholds.
Fernandes et al. 2017 [83]	Prospective cohort study	Patients with FS confirmed clinically and by imaging	n = 43	No comparison group; outcomes analysed by age, education, severity and number of nerve blocks	SSNB	Weekly SSNB using bupivacaine, continued until Constant–Murley score ≥55	From start to end of treatment	QoL and function improved significantly. Better outcomes were associated with older age, higher education, lower disease severity and fewer nerve blocks.
Galasso et al. 2023 [84]	Retrospective study with prospective data collection	Patients with FS resistant to conservative treatment	n = 78	Idiopathic, postoperative and posttraumatic FS	Arthroscopic capsular release	Patient-tailored arthroscopic release of contracted capsule/rotator interval; postoperative ROM and strengthening programme	Mean 54.2 months	Significant ROM and CMS improvement. High satisfaction. All patients returned to work/sport. Idiopathic aetiology predicted better postoperative CMS.
Romeo et al. 2023 [85]	Retrospective multivariable prognostic study	Patients with FS treated conservatively	n = 56	No formal treatment groups; analysed prognostic factors	Conservative treatment	Oral anti-inflammatory medication, home exercise or supervised physical therapy, and optional intra-articular steroid injection	≥1 year	PROMIS-UE, PROMIS Pain Interference, PROMIS Pain Intensity and VAS improved significantly. Anxiety, hyperlipidemia, higher BMI and Hispanic ethnicity were associated with less improvement. Female sex, manual labour and hypothyroidism were associated with better PROM changes.
Haroun et al. 2024 [86]	Prospective cohort study	Patients with FS > 3 months and failed conservative treatment	n = 57	Group 1: normal psychological status; Group 2: psychological distress by HADS ≥ 8	Arthroscopic capsular release	360° arthroscopic release + biceps tenotomy + postoperative rehabilitation	12 months	ROM and VAS pain improved significantly in all patients. Patients with anxiety/depression had higher pain preoperatively and at 12 months, but the magnitude of pain improvement was similar between groups.

Abbreviations: (FS): Frozen shoulder; (PRP): Platelet-Rich Plasma; (PN): Polynucleotide; (VAS): Visual Analogue Scale; (DASH): Disabilities of the Arm, Shoulder and Hand; (SST): Simple Shoulder Test; (ROM): Range of motion; (QoL): Quality of life; (SPADI): Shoulder Pain and Disability Index; (SSNB): Suprascapular nerve block; (HD): Hydrodistension; (PT): Institution-based physical therapy; (BC): Breast Cancer; (MUA): Manipulation under anaesthesia; (ISI): Intra-articular steroid injection; (NPRS): Numeric Pain Rating Scale; (SLAP): Superior Labrum Anterior to Posterior; (LHBT): Shoulder pain between concurrent long head of the biceps tendon; (SF-36): 36-Item Short Form Health Survey; (OSS): Oxford Shoulder Score; (ASES): American Shoulder and Elbow Surgeons score; (KSS): Korean Shoulder Score; (IA): Intra-Articular Injection; (ASES): American Shoulder and Elbow Surgeons score; (MUC): Manipulation under ultrasound-guided cervical nerve root block; (HAG): Hydrodilatation plus acupotomy group; (HG): hydrodilatation group; (PROM): Passive range of motion; (CMS): Constant–Murley Score; (AE): Adverse events; (TXA): Tranexamic Acid; (PRF): Pulsed Radiofrequency; (SMI): Superb Microvascular Imaging; (PDUS): Power Doppler ultrasonography; (CHL): Coracohumeral ligament; (MRI): Magnetic resonance imaging; (SSV): Subjective Shoulder Value; (AP): Axillary pouch; CHL: coracohumeral ligament; (SGHL): Superior glenohumeral ligament; (LHBT): long head of the biceps tendon; (IGHL): inferior glenohumeral ligament; (SNP): Single nucleotide polymorphism; (IL-1B): interleukin-1 β; (MMP-3): Matrix metalloproteinase-3; (TGF-B1): transforming growth factor β; (GDF5): Growth differentiation factor 5; (ELISA): Enzyme-linked immunosorbent assay; (AST): Aspartate aminotransferase; (ALT): Alanine aminotransferase; (GGT): Gamma-glutamyl transferase; (TSH): Thyroid-stimulating hormone; (NRS): Numeric Rating Scale; (T2D): type 2 diabetes; (HbA1c): Glycated haemoglobin; (SAIS): Subacromial impingement syndrome; (PSQI): Pittsburgh Sleep Quality Index; (SANE): Single Assessment Numeric Evaluation; (BAI): Beck Anxiety Inventory; (BDI): Beck Depression Inventory; (WHOQoL-BREF): World Health Organization Quality of Life—Brief; (HADS): Hospital Anxiety and Depression Scale; (PSEQ-10): Pain Self-Efficacy Questionnaire-10; (TSK-11): Tampa Scale for Kinesiophobia-11; (PCS): Pain Catastrophizing Scale; (RSWT): Radial shock wave therapy; (BMI): Body mass index; (PGIC): Patient Global Impression of Change; MCID: minimal clinically important difference; (CMS): Constant–Murley Score; (PROMIS-UE): Patient-Reported Outcomes Measurement Information System—Upper Extremity; (PROMIS Pain Interference): Patient-Reported Outcomes Measurement Information System Pain Interference; (PROMIS Pain Intensity): Patient-Reported Outcomes Measurement Information System Pain Intensity.

## Data Availability

The original contributions presented in this study are included in the article. Further inquiries can be directed to the corresponding author.

## Supplementary Materials

The following supporting information can be downloaded at: https://www.mdpi.com/article/10.3390/jcm15176684/s1.
