# Supplementary material for: Development of a Multidomain Conceptual Framework for Frozen Shoulder: A Systematic Review Integrating Clinical, Biological, Psychological and Lifestyle-Related Contributors
Source: J Clin Med. 2026 Aug 28;15(17):6684. doi: 10.3390/jcm15176684 (PMC13566836; doi:10.3390/jcm15176684)
Supplement: Supplementary file 1 [file jcm-15-06684-s001.zip › Supplementary Files S1-S4.pdf]

Supplementary File S1

Table S1-1: Risk of bias assessment of non-randomized intervention studies using the ROBINS-I tool

Study	Confounding	Selection	Classification	Deviations	Missing Data	Outcome Measurement	Reporting	Overall
Jain 2023	?	?	+	+	?	?	?	?
Lychagin 2022	-	?	+	?	?	?	+	?
Barman 2021	-	?	+	+	?	?	?	-
Lee 2022	?	?	+	+	?	?	?	?
Song 2021	-	?	+	+	?	?	?	-
Li 2023 (CHL)	-	?	+	+	?	?	?	-
Inglese 2024	?	?	+	+	?	?	?	?
Yildiz 2018	-	?	+	+	?	?	?	-
Martens 2023	-	?	+	+	?	?	?	-
Bongiorno 2023	-	?	+	+	?	?	?	-
Zhou 2025	-	?	+	+	?	?	?	-
Vastamäki 2016	?	?	+	+	?	?	?	?
Liu 2025	-	?	+	+	?	?	?	-
Pasqualini 2024	?	?	+	+	?	?	?	?
Luo 2025	-	?	+	+	?	?	?	-

Study	Confounding	Selection	Classification	Deviations	Missing Data	Outcome Measurement	Reporting	Overall
Kumar 2017	?	?	+	+	?	?	?	?
Dakkak 2024	-	?	+	+	?	?	?	-
Mertens 2022	-	?	+	+	?	?	?	-
Lesevic 2021	-	?	+	+	?	?	?	-
Li 2024	-	?	+	+	?	?	?	-
Kim 2021	-	?	+	+	?	?	?	-
Mardani-Kivi 2021	-	?	+	+	?	?	?	-
Mert 2025	-	?	+	+	?	?	?	-
Xu 2024	-	?	+	+	?	?	?	-
Albana 2022	?	?	+	+	?	?	?	?
Menekse 2024	-	?	+	+	?	?	?	-
Sivacioglu 2025	-	?	+	+	?	?	?	-
Haider 2022	-	?	+	+	?	?	?	-
Jung 2019	?	?	+	+	?	?	?	?
Atici 2021	-	?	+	+	?	?	?	-
Saito 2023	?	?	+	+	?	?	?	?
Wang 2024	?	?	+	+	?	?	?	?

Study	Confounding	Selection	Classification	Deviations	Missing Data	Outcome Measurement	Reporting	Overall
Satora 2021	-	?	+	+	?	?	?	-
Shang 2025	-	?	+	+	?	?	?	-
Yuan 2018	-	?	+	+	?	?	?	-
Bai 2023	-	?	+	+	?	?	?	-
Kim 2019	-	?	+	?	?	?	+	-
Wu 2025	-	?	+	?	?	?	+	-
Liang 2025	-	?	+	?	?	?	+	-
Dimitri-Pinheiro 2023	-	?	+	?	?	?	+	-
Galasso 2023	-	-	+	?	?	?	+	-

Risk of bias was assessed using the ROBINS-I tool across seven domains: bias due to confounding, selection of participants, classification of interventions, deviations from intended interventions, missing data, measurement of outcomes, and selection of the reported result. Each domain was rated as low (green), moderate (yellow), serious (red), or critical risk of bias. The overall risk of bias corresponds to the highest level of risk identified in any domain. Confounding was considered a key domain given the observational nature of the included studies.

Table S1-2: Risk of bias assessment of prognostic studies using the QUIPS tool.

Study	Participation	Attrition	Prognostic Factor	Outcome	Confounding	Analysis	Overall
Tache-Codreanu 2025	?	+	+	?	?	?	?
Fernandes 2017	?	?	?	?	-	?	-

Study	Participation	Attrition	Prognostic Factor	Outcome	Confounding	Analysis	Overall
Romeo 2022	+	+	+	+	?	+	?
Haroun 2024	+	+	+	+	?	+	?
Takahashi 2025	?	?	?	?	-	?	-
Takahashi 2024	?	?	?	?	-	?	-
Saito 2021	?	?	+	?	-	?	-
Dimitri-Pinheiro 2022	?	?	+	?	?	?	?

Risk of bias in prognostic studies was assessed using the QUIPS tool, which evaluates six domains: study participation, study attrition, prognostic factor measurement, outcome measurement, confounding, and statistical analysis and reporting. Each domain was rated as low (green), moderate (yellow), or high (red) risk of bias. The overall risk of bias was determined based on the presence and severity of bias across domains, with particular emphasis on confounding and adequacy of statistical adjustment.

Table S1-3: Methodological quality assessment of observational and cross-sectional studies using NIH tool.

Study	Selection	Measurement	Confounding	Analysis	Overall
Hamed 2024	+	+	?	+	?
Chen 2017	?	+	-	?	-
Tang 2024	?	+	?	?	?
Gillet 2021	+	+	?	+	?
Choi 2020	?	+	?	?	?
Kim 2019 (microflow)	?	+	?	?	?
Kim 2018	?	+	?	?	?
Stella 2022	?	+	?	+	?
Park 2016	?	+	?	?	?

Methodological quality of observational, cross-sectional, imaging, and descriptive studies was assessed using NIH and JBI tools, depending on study design. The evaluation

included domains such as participant selection, measurement of exposure and outcomes, identification and control of confounding factors, and appropriateness of statistical analysis. Risk of bias was categorized as low (green), moderate (yellow), or high (red). Due to the cross-sectional nature of many studies, confounding and lack of temporal relationship were considered major sources of bias.

Table S1-4: Methodological quality assessment of case reports using the JBI critical appraisal checklist.

**Study**	**Inclusion**	**Participants**	**Exposure**	**Outcome**	**Confounding**	**Strategies**	**Analysis**	**Overall**
**Mulligan 2015**	+	+	?	?	-	-	?	-
**Khan 2025**	?	?	?	?	-	-	?	-
**Fonseca 2025**	+	+	+	?	?	?	+	?
**Bhagade 2018**	?	?	+	?	-	-	?	-
**Toprak 2019**	+	+	?	+	+	-	+	?

Supplementary File S2

Domain assignment, predictor reassignment rules, and weighting structure

Note. Predictors were assigned according to their principal biological or clinical meaning rather than according to the nominal focus of the source paper, in order to reduce double counting across domains.

Table S2A. Domain assignment and predictor reassignment rules.

Predictor or construct	Final domain	Rationale for assignment
Baseline pain intensity	Clinical	Pain is a presentation-level symptom burden variable and forms part of the core clinical phenotype.
Symptom duration	Clinical	Duration reflects temporal clinical burden and disease-stage evolution rather than a separate biological domain.
Range-of-motion restriction	Clinical	External rotation, forward flexion, and abduction deficits are primary mechanical features of frozen shoulder presentation.
Stage / irritability	Clinical	Reflects clinical phase and symptom behaviour.
Disability / shoulder function	Clinical	Functional limitation is a direct manifestation of clinical burden.
Autonomic symptoms	Clinical	Retained as part of the clinical phenotypic expression rather than as a separate sphere.
Age, sex, BMI (contextual use)	Clinical	Retained as low-weight contextual covariates to preserve interpretability while avoiding overexpansion of separate domains.
Diabetes mellitus	Metabolic/endocrine	Assigned according to biological meaning as a major endocrine-metabolic comorbidity and strong adverse prognostic signal.
HbA1c	Metabolic/endocrine	Direct marker of glycaemic burden and metabolic severity.
Fasting glucose / glycaemic markers	Metabolic/endocrine	Represent graded metabolic dysregulation rather than clinical presentation.
Thyroid disease / thyroid dysfunction	Metabolic/endocrine	Endocrine comorbidity assigned according to biological mechanism.
TSH and thyroid-axis biomarkers	Metabolic/endocrine	Direct endocrine markers more appropriately grouped with metabolic/endocrine burden.
Dyslipidaemia / lipid abnormalities	Metabolic/endocrine	Reflect systemic metabolic risk rather than a primary clinical or imaging construct.
Liver-associated metabolic markers (e.g., GGT, AST, ALT)	Metabolic/endocrine	Retained as exploratory metabolic-biomarker signals within the endocrine-metabolic sphere.
MRI structural capsular abnormalities	Imaging	Structural tissue-level markers relevant to phenotype and prognosis.
Ultrasound structural capsular abnormalities	Imaging	Convergent with MRI structural findings and therefore retained within a unified imaging sphere.
Edema / hyperintensity / enhancement-related findings	Imaging	Represent activity-related tissue imaging behaviour rather than an isolated inflammatory sphere input.
Microvascular flow imaging	Imaging	Imaging-based vascular activity marker best interpreted within the imaging domain.
Compensatory sonographic features	Imaging	Tissue-level supportive phenotype markers retained within imaging.
Anxiety	Psychological	Core distress-related construct influencing pain burden and perceived recovery.
Depression	Psychological	Core mood-related construct within the psychological sphere.
Psychological distress (e.g., HADS-defined)	Psychological	Integrated measure of mood burden within the psychological domain.
Psychosocial quality of life	Psychological	Represents broader psychological and social burden.
Pain self-efficacy	Psychological	Reflects cognitive-affective recovery interference rather than clinical severity alone.
Kinesiophobia	Psychological	Recovery-related psychological modifier.
Catastrophising	Psychological	Psychological amplification construct.
Sleep-related burden / sleep disturbance	Psychological	Retained within the psychological sphere because of strong overlap with distress, pain-related burden, irritability, and psychosocial functioning.
IL-1beta and related pro-inflammatory markers	Inflammatory	Direct inflammatory biology retained in the inflammatory sphere; in the retained direct

		observational evidence, the principal signal was IL-1beta-related biomarker and susceptibility evidence.
IL-10 and resolution-related markers	Inflammatory	Represent anti-inflammatory/resolution biology; retained primarily as contextual or mechanistic support unless directly measured in a research dataset.
CRP / systemic inflammatory markers	Inflammatory	Reflect systemic inflammatory activity; retained as exploratory biological support.
HMGB1 / IL-33 / alarmins	Inflammatory	Exploratory inflammatory signalling markers retained as contextual biological constructs.
Inflammation-fibrosis bridge markers	Inflammatory	Retained as provisional inflammatory-biological bridge constructs linking cytokine activity, matrix remodelling, and capsular fibrosis.

Table S2B. Provisional within-domain weights.

Domain	Subdomain	Provisional weight (%)
Clinical	Baseline stiffness / range-of-motion restriction	30
Clinical	Symptom duration	22
Clinical	Autonomic symptoms	16
Clinical	Baseline pain	12
Clinical	Stage / irritability	8
Clinical	Baseline disability / function	7
Clinical	Contextual variables	5
Metabolic/endocrine	Diabetes-related burden	36
Metabolic/endocrine	Glycaemic control	26
Metabolic/endocrine	Thyroid / endocrine-axis variables	18
Metabolic/endocrine	Lipid metabolism	12
Metabolic/endocrine	Liver-associated metabolic markers	8
Imaging	Capsular structural biomarkers	44
Imaging	Edema / hyperintensity / activity-related signs	28
Imaging	Microvascular imaging	18
Imaging	Compensatory sonographic features	10
Psychological	Distress / mood	34
Psychological	Sleep-related burden	28
Psychological	Psychosocial quality of life	22
Psychological	Psychological interference with perceived recovery	16
Inflammatory	Pro-inflammatory cytokine signals	34
Inflammatory	Anti-inflammatory / resolution signals	22
Inflammatory	Systemic low-grade inflammation	18
Inflammatory	Alarmin signalling	14
Inflammatory	Inflammation-fibrosis bridge	12

Note. Within each domain, provisional subdomain weights were normalised to sum to 100%. The inflammatory domain includes the inflammation-fibrosis bridge subdomain so that its internal weights sum to 100%.

Table S2C. Provisional cross-domain weights and four-domain sensitivity specification.

Domain	Five-domain model weight (%)	Four-domain sensitivity weight (%)	Interpretation
Clinical	21.1	24.0	Retained as a major presentation-level domain.
Metabolic/endocrine	24.0	27.3	Highest provisional cross-domain contribution.
Imaging	22.9	26.0	High contribution driven by structural and activity-related biomarkers.
Psychological	20.0	22.7	Substantial contribution including distress/mood and sleep-related burden.
Inflammatory	12.0	Omitted	Retained in the five-domain model as provisional, lower-confidence support; omitted in the sensitivity specification.

Note. The four-domain sensitivity specification omits the inflammatory domain and renormalises the remaining domains to 100%. It is intended to examine stability of the framework when the least-supported domain is removed, not to validate the model.

Supplementary File S3

Main evidence signals supporting sphere interpretation and weighting

Note. This table summarises dominant evidence patterns used to support domain interpretation and provisional weighting. It does not replace study-level detail and should be interpreted alongside the risk-of-bias assessment and weighting matrix.

Table S3. Main quantitative signals supporting each domain.

Domain	Main quantitative signals supporting domain
Clinical	The clinical domain was primarily supported by repeated signals linking baseline stiffness/range-of-motion restriction and symptom duration with outcome trajectory and prognosis. Across the retained literature, pain improved earlier than mobility, while gains in range of motion continued over later follow-up windows. Baseline pain, disability, and stage/irritability provided secondary support, but range-of-motion restriction and symptom duration remained the dominant recurrent clinical signals.
Metabolic/endocrine	The metabolic/endocrine domain was chiefly supported by recurrent diabetes-related and glycaemic-control signals. Diabetes mellitus repeatedly emerged as an adverse factor associated with poorer treatment response, recurrence, reintervention, or worse long-term outcome. Glycaemic-control variables, particularly HbA1c and glucose-related measures, reinforced this signal by suggesting that metabolic severity extends beyond binary diabetes status. Thyroid/endocrine-axis variables contributed a secondary but relevant signal, while dyslipidaemia and liver-associated metabolic markers provided additional exploratory support.
Imaging	The imaging domain was supported mainly by structural capsular biomarkers and activity-related imaging signs. Coracohumeral ligament thickening, capsular thickening, inferior glenohumeral ligament-related abnormalities, and axillary recess findings repeatedly correlated with reduced motion, disease phenotype, or prognosis. Edema-, hyperintensity-, and enhancement-related findings contributed a second layer consistent with activity or stage. Microvascular imaging findings provided additional support for a dynamic tissue-activity component, while compensatory sonographic features were retained as a smaller supportive layer.
Psychological	The psychological domain was supported by repeated evidence linking distress/mood-related variables with pain burden and recovery, and by sleep-related studies showing associations with disability, irritability, quality of life, and overall symptom burden. Distress/mood remained the strongest psychological signal, but sleep-related burden emerged as the second major component after targeted review of the frozen shoulder sleep literature. Psychosocial quality of life and recovery-interference constructs, including self-efficacy and kinesiophobia, provided additional but smaller support.
Inflammatory	The inflammatory domain was retained on the basis of direct but limited biomarker/susceptibility evidence and broader biological plausibility. The principal retained direct signal was centred on IL-1beta-related evidence, including higher serum IL-1beta in primary frozen shoulder and an IL-1beta polymorphism associated

	with susceptibility. Other inflammatory constructs, including resolution markers, systemic inflammatory markers, alarmins, and inflammation-fibrosis bridge mechanisms, were treated as contextual or mechanistic support rather than as evidence of a mature observational weighting base. Accordingly, this domain was interpreted as biologically relevant but quantitatively provisional and lower confidence.

Supplementary File S4

Prototype operational input matrix

Note. The matrix summarises preferred patient-level inputs, scale types, severity direction, and operational status for the research-use prototype. The prototype is exploratory and has not been validated as a clinical decision-support tool.

Table S4A. Prototype operational input matrix.

Domain	Subdomain	Preferred operational input(s)	Preferred scale / format	Severity direction	Operational status
Clinical	Baseline stiffness / range-of-motion restriction	External rotation deficit, forward flexion deficit, abduction deficit	Degrees, preferably expressed as deficit vs contralateral side or expected normative value	Higher deficit = greater burden	Core operational input
Clinical	Symptom duration	Symptom duration	Months since symptom onset	Longer duration = greater burden	Core operational input
Clinical	Autonomic symptoms	Autonomic symptom burden	0-10 clinical rating or rescaled questionnaire score	Higher score = greater burden	Supportive input
Clinical	Baseline pain	Pain intensity	VAS/NRS 0-10	Higher score = greater burden	Core operational input
Clinical	Stage / irritability	Clinical stage or irritability	Ordinal clinical classification	Higher stage/irritability = greater burden	Supportive input
Clinical	Baseline disability / function	Shoulder disability	SPADI total	Higher score = greater burden	Core operational input
Clinical	Contextual variables	Age, sex, BMI	Demographic/contextual variables	Context-dependent	Low-weight contextual input
Metabolic/endocrine	Diabetes-related burden	Diabetes mellitus	Binary yes/no	Presence = greater burden	Core operational input
Metabolic/endocrine	Glycaemic control	HbA1c, fasting glucose	Continuous biomarker values	Higher abnormality = greater burden	Core operational input
Metabolic/endocrine	Thyroid / endocrine-axis variables	Thyroid disease, TSH abnormality	Binary and/or biomarker-based	Greater abnormality = greater burden	Supportive input
Metabolic/endocrine	Lipid metabolism	Dyslipidaemia, lipid abnormality	Binary and/or biomarker-based	Presence/abnormality = greater burden	Supportive input
Metabolic/endocrine	Liver-associated metabolic markers	GGT, AST/ALT-related abnormality	Biomarker-based	Greater abnormality = greater burden	Exploratory/supportive input
Imaging	Capsular structural biomarkers	CHL thickness, axillary recess thickness, IGHL-related structural findings	MRI and/or ultrasound	Greater structural abnormality = greater burden	Core imaging input
Imaging	Edema / hyperintensity / activity-related signs	Edema, hyperintensity, enhancement-related findings	Semi-quantitative MRI/US rating	Greater activity = greater burden	Core/supportive imaging input
Imaging	Microvascular imaging	Vascular flow signal	SMI/Doppler-type metric	Greater vascular activity = greater burden	Supportive imaging input
Imaging	Compensatory sonographic features	Supportive compensatory findings	Ultrasound feature composite	Greater compensatory signal = greater burden	Exploratory/supportive input
Psychological	Distress / mood	Anxiety and depression burden	HADS-based constructs	Higher score = greater burden	Core psychological input
Psychological	Sleep-related burden	Sleep disturbance / sleep quality	PSQI	Higher score = greater burden	Core psychological input
Psychological	Psychosocial quality of life	Psychological quality-of-life deficit	WHOQOL-BREF psychological domain or equivalent	Lower QoL = greater burden	Supportive input
Psychological	Psychological interference with perceived recovery	Self-efficacy deficit, kinesiophobia	PSEQ, TSK or equivalent	Greater interference = greater burden	Supportive input
Inflammatory	Pro-inflammatory cytokine signals	IL-1beta-related biomarker activity; optional cytokine composite in	Biomarker-based	Greater abnormality = greater burden	Provisional/research-only input

		research datasets			
Inflammatory	Anti-inflammatory / resolution signals	IL-10 or related resolution markers	Biomarker-based	Lower resolution activity = greater burden	Contextual/research-only input
Inflammatory	Systemic low-grade inflammation	CRP or comparable marker	Biomarker-based	Greater abnormality = greater burden	Exploratory/research-only input
Inflammatory	Alarmin signalling	HMGB1 / IL-33-related markers	Biomarker-based	Greater abnormality = greater burden	Contextual/research-only input
Inflammatory	Inflammation-fibrosis bridge	Composite biological bridge marker	Composite biomarker-based	Greater abnormality = greater burden	Contextual/research-only input

Table S4B. Standardisation and missing-data rules used in the research-use prototype.

Component	Prototype rule
Binary variables	Coded as 0 or 100 according to absence or presence of the burden-related feature.
Continuous variables	Linearly rescaled to predefined ranges for prototype calculation. This scaling is operational only and does not imply that biological relationships are linear.
Ordinal variables	Mapped to equally spaced 0-100 categories.
Range-of-motion variables	Converted to movement deficits so that higher values consistently represent greater burden.
Partially missing input within a subdomain	Available inputs within the same subdomain are proportionally reweighted.
Missing entire subdomain	Remaining subdomains within the corresponding domain are rescaled to sum to 1, with reduced-confidence interpretation.
Inflammatory domain unavailable	A four-domain sensitivity specification may omit the inflammatory domain and renormalise clinical, metabolic/endocrine, imaging, and psychological weights to 100%.
Worked example	Any numerical score generated by the prototype is illustrative only and should not be interpreted as a validated diagnostic, prognostic, or treatment-allocation threshold.

Note. These rules were used to demonstrate operational feasibility and should be interpreted as methodological assumptions requiring future validation, calibration, and stability testing in prospective datasets.
